# Structural Insights into the PorK and PorN Components of the *Porphyromonas gingivalis* Type IX Secretion System

**DOI:** 10.1371/journal.ppat.1005820

**Published:** 2016-08-10

**Authors:** Dhana G. Gorasia, Paul D. Veith, Eric G. Hanssen, Michelle D. Glew, Keiko Sato, Hideharu Yukitake, Koji Nakayama, Eric C. Reynolds

**Affiliations:** 1 Oral Health CRC, Melbourne Dental School, Bio21 Institute, The University of Melbourne, Melbourne, Victoria, Australia; 2 Melbourne Advanced Microscopy Facility and Department of Biochemistry and Molecular Biology, Bio21 Molecular Science and Biotechnology Institute, The University of Melbourne, Parkville, Victoria, Australia; 3 Division of Microbiology and Oral Infection, Department of Molecular Microbiology and Immunology, Nagasaki University Graduate School of Biomedical Sciences, Nagasaki, Japan; Osaka University, JAPAN

## Abstract

The type IX secretion system (T9SS) has been recently discovered and is specific to *Bacteroidetes* species. *Porphyromonas gingivalis*, a keystone pathogen for periodontitis, utilizes the T9SS to transport many proteins including the gingipain virulence factors across the outer membrane and attach them to the cell surface via a sortase-like mechanism. At least 11 proteins have been identified as components of the T9SS including PorK, PorL, PorM, PorN and PorP, however the precise roles of most of these proteins have not been elucidated and the structural organization of these components is unknown. In this study, we purified PorK and PorN complexes from *P*. *gingivalis* and using electron microscopy we have shown that PorN and the PorK lipoprotein interact to form a 50 nm diameter ring-shaped structure containing approximately 32–36 subunits of each protein. The formation of these rings was dependent on both PorK and PorN, but was independent of PorL, PorM and PorP. PorL and PorM were found to form a separate stable complex. PorK and PorN were protected from proteinase K cleavage when present in undisrupted cells, but were rapidly degraded when the cells were lysed, which together with bioinformatic analyses suggests that these proteins are exposed in the periplasm and anchored to the outer membrane via the PorK lipid. Chemical cross-linking and mass spectrometry analyses confirmed the interaction between PorK and PorN and further revealed that they interact with the PG0189 outer membrane protein. Furthermore, we established that PorN was required for the stable expression of PorK, PorL and PorM. Collectively, these results suggest that the ring-shaped PorK/N complex may form part of the secretion channel of the T9SS. This is the first report showing the structural organization of any T9SS component.

## Author Summary

Chronic periodontitis is a major public health problem and *Porphyromonas gingivalis* is considered a keystone pathogen of the disease. The major virulence factors of *P*. *gingivalis* are the extracellular proteases called the gingipains. These gingipains and 30 other proteins that have a conserved C-terminal domain are transported across the outer membrane via a recently discovered secretion system designated the type IX secretion system (T9SS). To date, at least 11 proteins including PorK, PorL, PorM and PorN have been identified to be components of the T9SS, however it is unknown which proteins comprise the actual secretion channel in the outer membrane. Here we demonstrate that PorK and PorN form a large ring-shaped structure that is localised in the periplasm and is very likely to form part of the T9SS channel. Additionally, we show that PorN is required for the stable production of PorK, PorL and PorM and that PorL and PorM form a separate stable complex. This is the first study to show the structural organisation of T9SS components.

## Introduction

Pathogenic Gram-negative bacteria have evolved very complicated machineries to secrete virulence factors which aid in infection and colonization of their hosts. To date, nine different types of secretion systems have been identified in bacteria [1, 2]. Most of these secretion systems form large complexes consisting of multiple components and high resolution structures of assembled type III and IV secretion systems have been obtained using cryo-electron microscopy [3, 4].

The type IX secretion system (T9SS), formerly designated the Por secretion system, has been recently identified and is conserved in many species of the *Bacteroidetes* phylum, which is a diverse group of Gram-negative bacteria [5–8]. The substrates of the T9SS have a signal peptide at the N-terminus which directs the passage across the cytoplasmic membrane via the SEC machinery and also contains a conserved C-terminal secretion signal that allows the translocation across the outer membrane (OM). After translocation the secretion signal is cleaved and the substrates undergo extensive glycosylation which allows their attachment to the cell surface [7, 9].

The bacterial pathogen *Porphyromonas gingivalis* is an anaerobic Gram-negative bacterium that has been highly associated with chronic periodontitis [10]. *P*. *gingivalis* secretes extracellular and cell-surface cysteine proteinases called gingipains via the T9SS. Gingipains (RgpA, RgpB and Kgp) are the major virulence factors of this organism [11]. In addition to the gingipains a total of 30 other proteins, including the virulence factors CPG70, peptidylarginine deiminase (PAD) and hemin binding protein (HBP35), were identified as T9SS substrates [12–16].

Multiple components of the T9SS have been identified through characterization of the respective secretion deficient mutants in *P*. *gingivalis*. PorT was the first identified component of the T9SS and subsequently ten additional proteins, namely PorK, PorL, PorM, PorN, PorP, PorQ, PorU, PorV, PorW, and Sov were shown to be required for the secretion of gingipains [17]. All the mutants involved in the T9SS were found to cause an accumulation of precursor gingipains in the periplasm and had considerably reduced gingipain activity on the cell surface [8, 18–22]. PorK, PorL, PorM, PorN, PorW, PorT, and Sov share similarity in amino acid sequence with gliding motility proteins GldK, GldL, GldM, GldN, SprE, SprT and SprA, respectively, in *Flavobacterium johnsoniae*, suggesting that the T9SS is linked to the gliding motility of bacteria in the *Bacteriodetes* phylum [8, 23]. Deletion of *gldK*, *gldL*, *gldM*, *gldN*, *sprE*, *sprT* and *sprA* in *F*. *johnsoniae* have been shown to cause defects in gliding motility indicating that these proteins are required for the secretion of cell surface motility adhesins SprB and RemA [8, 23–25].

Although several proteins have been identified to affect the secretion of gingipains and other CTD proteins, the role of most of these proteins remain unknown. To date specific functions have only been assigned to PorV (LptO) and PorU (PG0026). LptO has been shown to be essential for the O-deacylation of LPS lipid A [22] and PG0026 has been demonstrated to be the CTD peptidase that cleaves the CTD (the C- terminal secretion signal) on the cell surface [26] and more recently it has been proposed to be the transpeptidase that cleaves the CTD and attaches A-LPS to the new C-terminus of the T9SS substrates in a single reaction [9]. However, nothing is known on how the T9SS components organize to form a secretion channel that allows the transport of the CTD proteins across the outer membrane.

A study by Sato et al. suggested that PorK, PorL, PorM and PorN together form a complex greater than 1200 kDa by blue native PAGE analysis [8]. We hypothesized that PorK, PorL, PorM and PorN form part of the secretion channel of the T9SS. In this study, we demonstrate that PorK and PorN form a very large ring-shaped structure that is associated with the periplasmic face of the outer membrane and is likely to be associated with the type IX secretion channel. We also show that PorL and PorM form a separate stable complex apart from PorK and PorN. Together, this is the first study showing the structural organization of components of the T9SS.

## Results

### Lack of EDSL on *porK*, *porL*, *porM* and *porN* mutant cells

**Fig 1:**
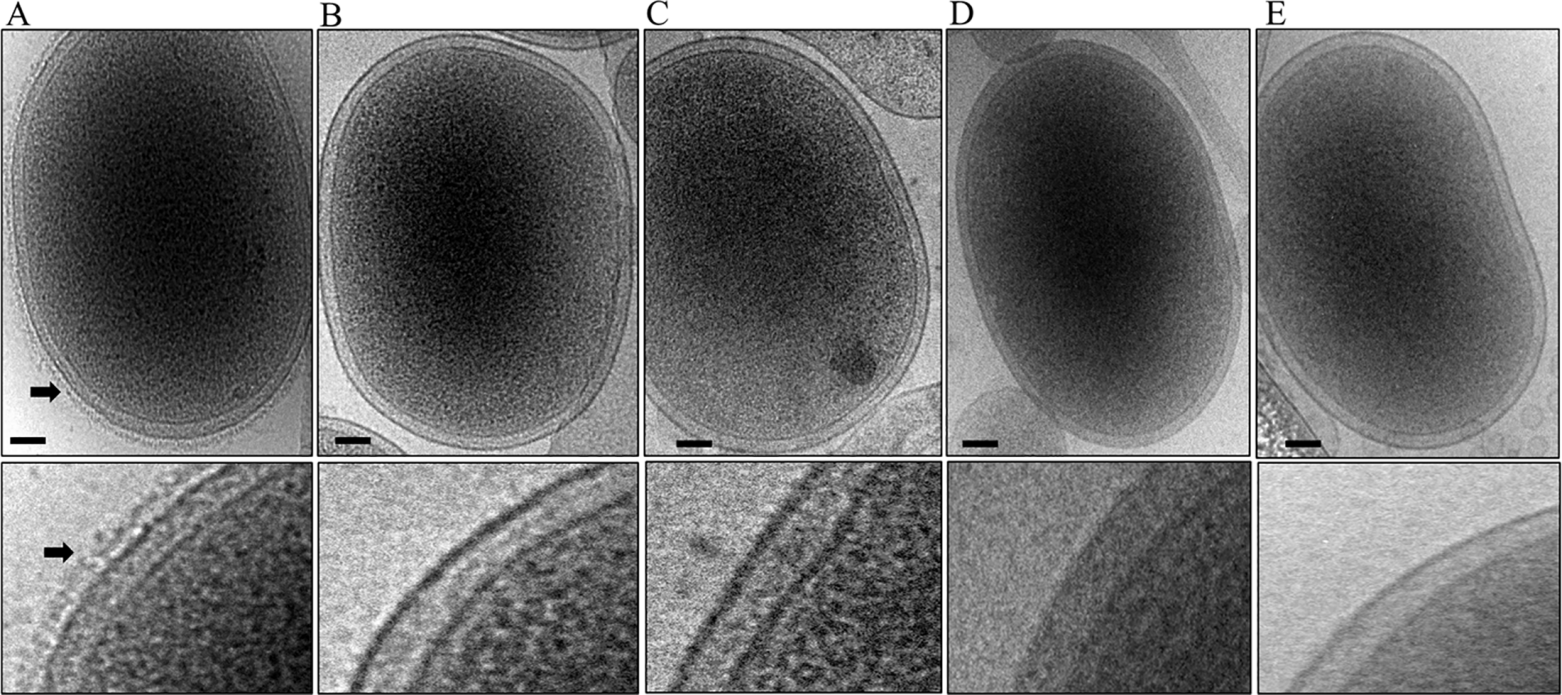
Absence of EDSL on *porK*, *porN*, *porL* and *porM* mutants. Cryo-EM micrographs of whole cells (A) Wild type (B) *porK* mutant (C) *porN* mutant (D) *porL* mutant and (E) *porM* mutant. A magnified section is shown on the bottom of each image. Arrows point to the electron-dense surface layer (EDSL) that is absent in the mutants. Scale bars 100 nm.

In wild type *P*. *gingivalis* the gingipains and other CTD proteins are secreted and attached to the surface of the cell producing an electron-dense surface layer (EDSL) (**Fig 1A**) [9, 22]. Mutants that are defective in the T9SS, such as the LptO and PG0026 mutants, lack the EDSL [22, 26]. Sato et al. have shown that the absence of PorK, PorL, PorM and PorN proteins in *P*. *gingivalis* causes severe defects in the secretion of gingipains to the cell surface [8]. To investigate if the EDSL is affected by the lack of PorK, PorL, PorM and PorN, cryo-EM was performed on *porK*, *porL*, *porM* and *porN* mutant cells. As expected no visible EDSL was noted in any of these mutants (**Fig 1**), confirming the importance of these four genes in the T9SS.

### Isolation of PorK and PorN complex from *P*. *gingivalis*

**Fig 2:**
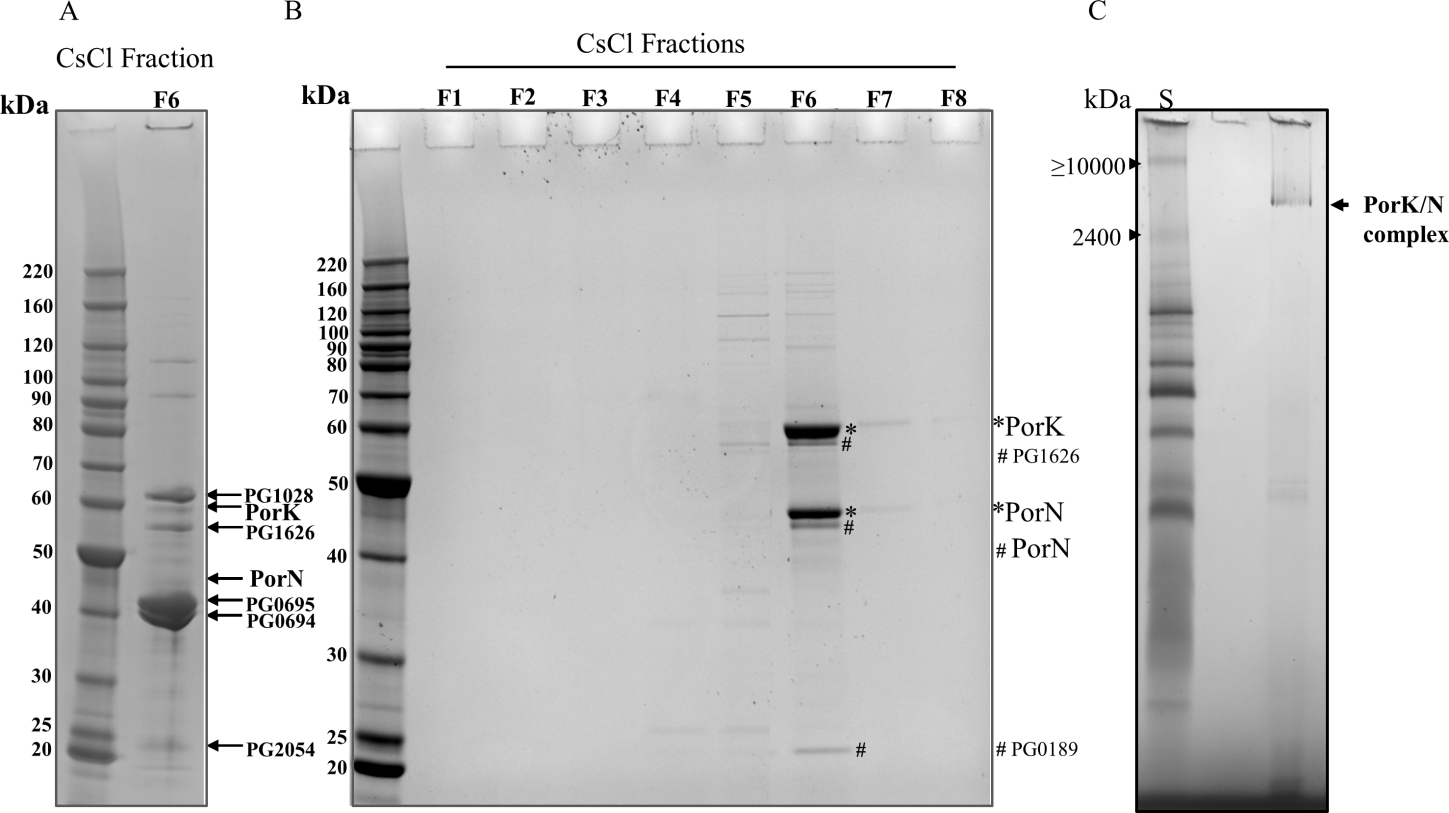
PorK/N complex isolated from *P*.*gingivalis*. (A) An attempt at the purification of PorKLMN complexes from W50ABK*WbaP using a published method (see methods section). All fractions from CsCl density gradients were resolved by SDS-PAGE and only fraction 6 had protein bands which is shown in the figure. Indicated bands were identified by mass spectrometry. (B) The PorK and PorN complex was purified from W50ABK*WbaP using a modified protocol (see methods section). Fractions from CsCl density gradients were resolved by SDS-PAGE and visualized by Coomassie stain. PorK and PorN were identified by mass spectrometry (Table 1). (C) Blue native PAGE analysis of purified PorK/N complexes. Lane S- is bovine heart mitochondria solubilized in 1% digitonin. The protein bands corresponding to molecular masses of ≥ 10000 kDa and 2400 kDa bands were identified by mass spectrometry as pyruvate dehydrogenase complex and the tetrameric form of ATP synthase, respectively.

To further investigate the role of these proteins in the secretion of CTD proteins, we attempted to purify the PorKLMN complex from the W50ABK*WbaP mutant. The rationale for selecting this strain over wild type was to avoid the presence of gingipains which are known to form large complexes and also to circumvent the digestion of the complexes of interest by the gingipains. Purification of cellular complexes is further simplified in this strain, since in the W50ABK*WbaP mutant, all CTD proteins are eliminated as they are secreted into the growth medium [9]. At first the method used for the purification of the Type III secretion system needle complex was utilized [3]. The major fraction isolated by this procedure (Fraction 6) contained major protein bands at about 40 kDa (**Fig 2A**). These bands were identified as Omp40 and Omp41, which are known to be OmpA-like proteins that bind to peptidoglycan [27]. Other strong bands at approximately 60 kDa and 20 kDa contained PG1028 and PG2054 respectively as their major constituents, and these proteins share the same OmpA-like peptidoglycan domain as found in Omp40 (PG0694) and Omp41 (PG0695). We therefore hypothesized that peptidoglycan polymers had survived the procedure and were cross-linking proteins into large complexes. PorK and PorN were also identified in this fraction, but were present in relatively small amounts (**Fig 2A**). Therefore the isolation method was modified by performing the peptidoglycan hydrolysis with lysozyme after rather than before cell lysis thereby increasing the exposure of the peptidoglycan (see Materials and Methods). A total of eight fractions were collected after CsCl gradient centrifugation and analysed by SDS-PAGE. Intense bands of two protein species at the molecular weights (MWs) of ~ 45 kDa and 60 kDa were observed in fraction number six together with other very less intense protein bands (**Fig 2B**). To identify the two intense protein bands, an in gel tryptic digestion was performed on the gel bands and the proteins were identified by mass spectrometry (MS) (**Table 1**). The 45 kDa band was identified as PorN (calculated mass = 39 kDa), and the 60 kDa protein band was identified as PorK (calculated mass = 53 kDa) (**Table 1, S1 Table**). The minor bands marked with # were identified as PG1626, PorN and PG0189 (**Fig 2B, S1 Table**). PorN may have been degraded to some degree during the purification process while PG1626 and PG0189 appeared to have been co-purified with the complex. The other minor bands were also identified (**S1 Table**). Of note, no PorL or PorM was identified in this fraction indicating PorK and PorN forms a stable complex without PorL or PorM.

**Table 1:** Identification of PorK and PorN protein bands by mass spectrometry.

Name	Accession No.	Mascot Score	Seq. Coverage (%)	No. of peptides
PorK	PG0288	1566	54	22
PorN	PG0291	815	46	14

### PorK and PorN form a complex of ≥ 4500 kDa

To determine the MW of the complex the purified PorK and PorN (PorK/N) complex was analysed by blue native PAGE. Since the highest MW in the commercial native protein ladder was 1200 kDa, we utilized bovine heart mitochondria lysate to estimate the MW of PorK/N complex. The highest two bands in the lysate were identified by MS to be pyruvate dehydrogenase complex (≥ 10000 kDa) and the tetrameric form of ATP synthase (2400 kDa). The purified complex migrated as a single band and the estimated molecular weight was ≥ 4500 kDa (**Fig 2C**). The protein band was verified to be PorK and PorN by MS (**S2 Table**).

### Ring-shaped structures of PorK and PorN complex

**Fig 3:**
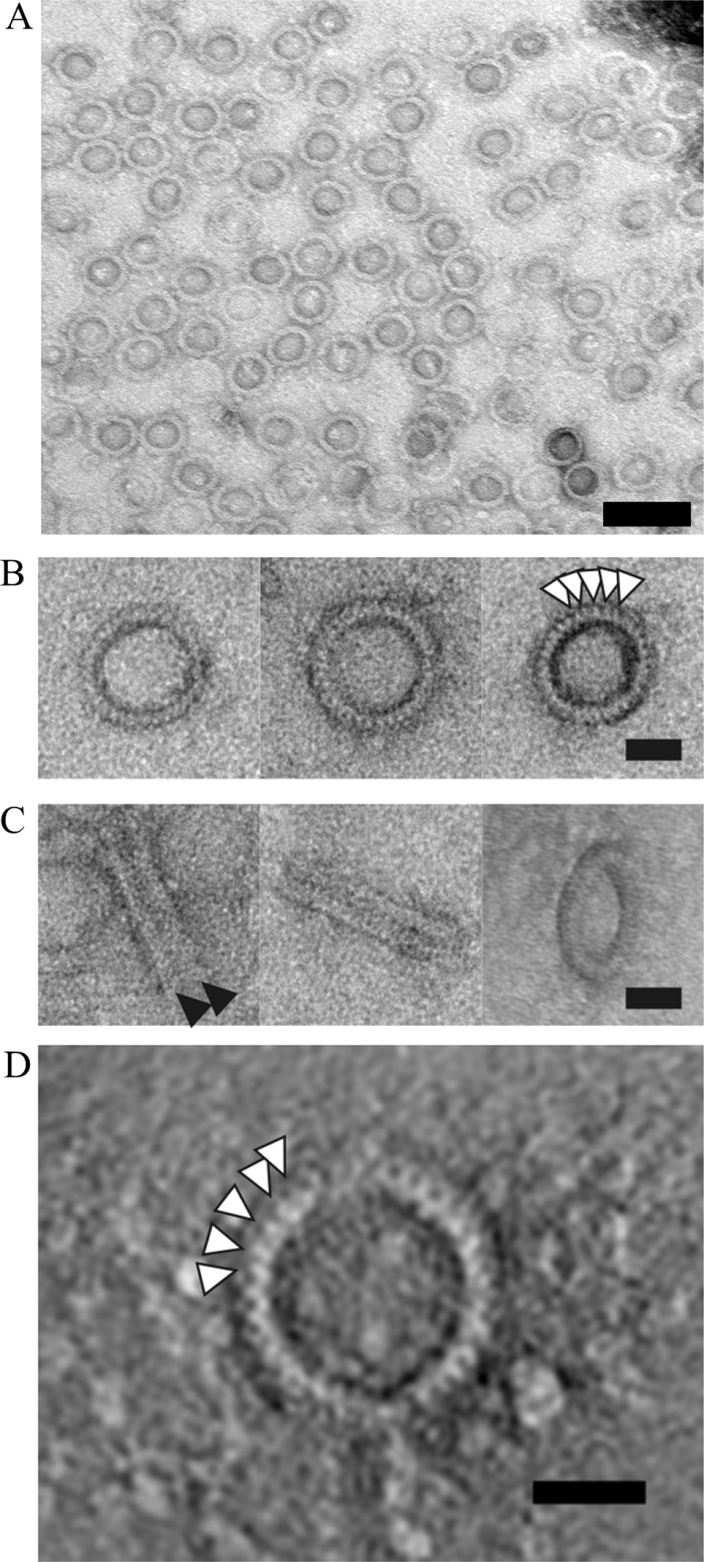
Electron micrographs of negatively stained PorK/N complex. Complexes in fraction 6 of CsCl density gradients were stained with uranyl acetate and observed under TEM. (A) Homogenous ring-shaped structures of PorK/N complex. Scale bar 100 nm (B) Top view of the complex forming a ring with 32–36 subunits (white arrow heads). Scale bar 20 nm. (C) The first two columns show side views of the complex where two major rings were observed (black arrowheads). Scale bar 20 nm. The third column shows the tilted view of the complex verifying the presence of two rings. (D) A higher resolution image obtained by virtual section (0.76 nm) through an electron tomogram of a negatively stained complex. At this resolution individual subunits (32) were observed. Scale bar 20 nm.

To explore the structure of the PorK/N complex, we performed electron microscopy and electron tomography on negatively stained complexes. A homogenous population of ring shaped structures was observed (**Fig 3A**). The rings consistently measured from 46–52 nm in diameter, with an internal pore size diameter of 33–35 nm. The rings were composed of 32–36 smaller subunits (**Fig 3B**). Side views suggested the presence of two rings with a combined thickness of 15 nm (**Fig 3C**). 3-D tomography of a single particle confirmed the appearance of 32 subunits in that particular particle (**Fig 3D**).

**Fig 4:**
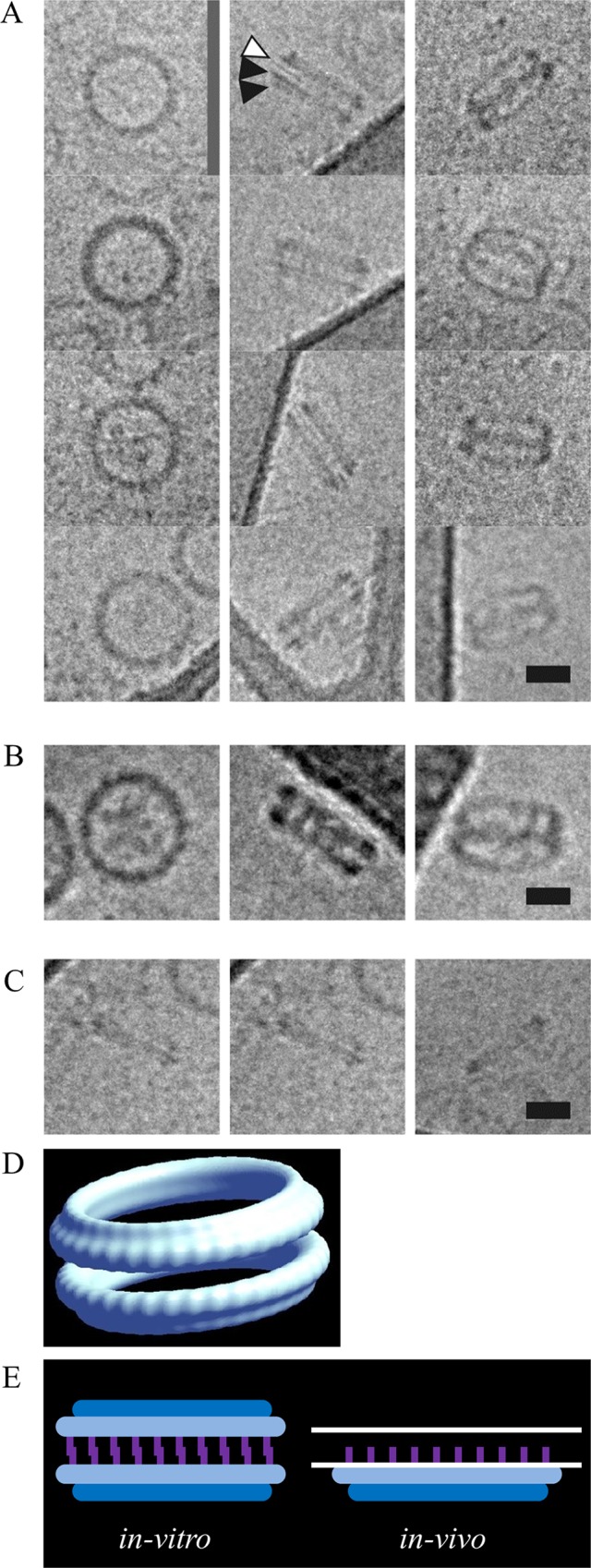
Cryo transmission electron micrographs of the purified PorK/N complex. (A) First column -top views of the complex, second column- side views, third column—tilted views. The side views show two major rings and within each major ring two distinct sub-rings were observed, shown by the black arrow heads. Note the thin electron dense band between the two major rings, shown by the white arrow head, which may represent the lipid tails overlap of the PorK lipoprotein. (B) Purified native PorK/N complex from the wild type has the same structure as from the mutant (W50ABK*WbaP) at that resolution. (C) LDAO treated complex, the association between the two major rings has been ablated and single complexes are now noticeable. Scale bars 20 nm. (D) A 3-D reconstruction of the PorK/N ring. (E) Schematic representations of the PorK/N rings. The double major ring form (*in-vitro*) is proposed to be an artifact of the two major rings interacting via the PorK lipid shown in purple. Whereas the *in-vivo* form is proposed to be a single major ring composed of PorN (dark blue) and PorK (light blue) with the PorK lipid (purple) involved in anchorage to the OM which is represented by two white parallel lines.

In order to verify the structure obtained from negatively stained samples, we performed cryo-electron microscopy, where the sample was viewed in its native form under cryogenic conditions (**Fig 4**). The side views obtained showed the presence of two major rings (**Fig 4A, middle column**) and closer inspection revealed that each of the major rings consisted of two distinct sub-rings, one sub-ring having a larger diameter than the other sub-ring, indicated by black arrow heads in the figure (**Fig 4A, middle column**). The larger sub-ring was always observed on the inside of the complexes. Additionally, a narrow band was observed between the two major rings, which may function to hold the complex together. In order to confirm that the observed complex was not an artifact of the mutant used, the PorK/N complex was also purified from the wild type strain. Very similar ring-shaped complexes were observed indicating that the complex purified from W50ABK*WbaP was the same as wild type (**Fig 4B**). When the purified complex from W50ABK*WbaP was resuspended in LDAO detergent instead of DDM detergent, the two major rings appeared to dissociate into single major rings (**Fig 4C**). It is this single major ring that we propose to be the biologically relevant structure (see **Fig 4E** and “Discussion”). **Fig 4D** shows a 3D-reconstruction of the PorK/N double ring in DDM. The resolution was above the cut off of 40.5 Å for it to be determined precisely using the gold standard Fourier Correlation Shell. In this structure two major rings were observed and within each major ring two sub-rings were clearly visualized. Although various conditions were trialed we were unable to obtain the very large number of individual particles required for a higher resolution 3D-structure due to the difficulties with aggregation of the sample and the preferred orientation on the carbon.

### PorK and PorN stoichiometric interaction is 1:1 in the complex

To determine the stoichiometry of PorK and PorN present in the complex we performed high sensitivity amino acid analysis on protein bands that were transferred on to the PVDF membrane. The molar ratio calculated for PorK:PorN was 1:1.2 suggesting that PorK and PorN may be present in cells in a 1:1 molar ratio (**S3 Table**). Although densitometry analysis of Coomassie stained gels (such as Fig 2B) showed that the PorK band was slightly larger / more intense than the combined PorN bands, the molar ratio ranged from 1:1 to 1:1.2 (PorK:PorN). Since both of these analyses depend on the purity of the bands, each band was digested with trypsin, analysed by Orbitrap LC-MS and the purity estimated by quantifying the proteins present with MaxQuant software. Using the LFQ metric, the PorK and PorN bands were estimated to be 94% and 95% pure respectively (**S4 Table**), validating the use of amino acid analysis and densitometry analysis to provide a reliable estimate of stoichiometry. Collectively, these results suggest that the stoichiometry of the PorK/N complex is 1:1.

### The PorK and PorN complex is anchored to the outer membrane and localized in the periplasm of *P*. *gingivalis*

**Fig 5:**
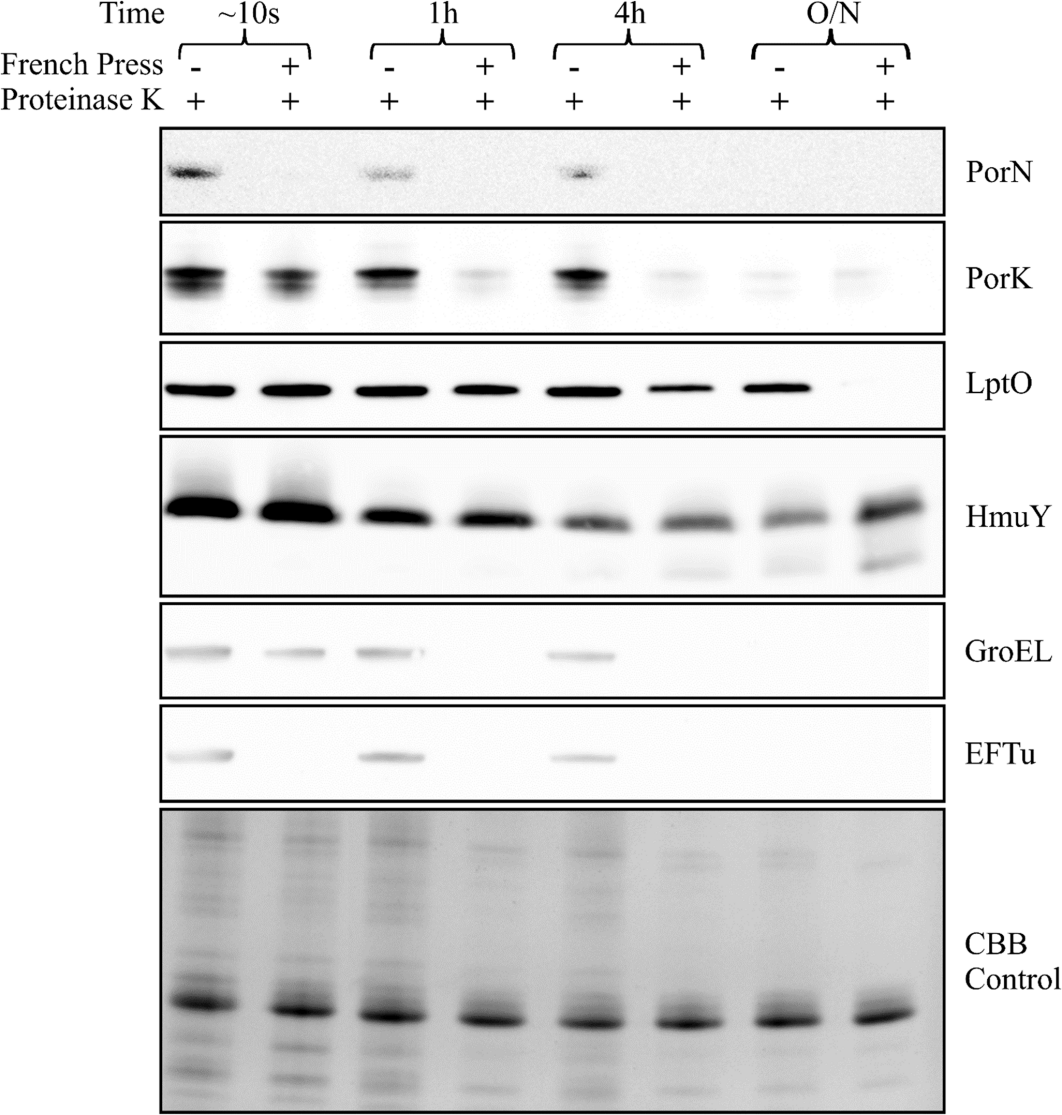
Determination of subcellular localization using proteinase K accessibility. *P*. *gingivalis* cells (W50ABK*WbaP) were either lysed using a French Pressure cell or were left unlysed. Cells were treated with proteinase K and aliquots were collected at 0 (10s), 1, 4 h and after overnight incubation. Samples were subjected to SDS-PAGE followed by immunoblot analysis using antisera against the protein indicated on the right. The Coomassie blue stained gel shows the relative loading amount.

Previously, PorK and PorN have been localized to the OM and PorK was also found to have a typical lipoprotein signal peptide suggesting it is an outer membrane lipoprotein [8]. To distinguish whether PorK and PorN are OM-embedded or peripheral OM proteins attached to either the outer side or periplasmic side of the OM, we used a proteinase K susceptibility assay. Whole cells and lysed cells were treated with proteinase K and aliquots taken at various time points were subjected to SDS-PAGE and immunoblot analyses. Anti-PorN and anti-PorK reactive bands were present in the fractions that were not lysed for up to 4 hours. However in the lysed fractions the PorN band was not observed at any time point and the PorK band was substantially diminished by 1h (**Fig 5**). LptO is an integral outer membrane protein [12, 22]. Anti-LptO reactive bands appeared in all fractions except for the lysed cells with overnight incubation. In addition, there was little difference in the intensity of the LptO bands between lysed and unlysed cells for up to 4h (**Fig 5**). HmuY is a peripheral outer membrane lipoprotein [28]. Anti-HmuY reactive bands again appeared in all fractions. There was no difference in the intensity of the band between lysed and unlysed cells at each time point, however there was a small decrease in the intensity of the HmuY band with time (**Fig 5)**. GroEL and elongation factor tu (EFtu) are cytoplasmic proteins and the immunoblot profile was similar to the PorK and PorN profiles, respectively (**Fig 5**). The extreme sensitivity of PorK and PorN to proteinase K after cell lysis together with their resistance to cleavage in whole cells are consistent with a location on the periplasmic side of the OM. The integral OM and surface proteins tested in contrast were very resistant to proteolysis consistent with their natural exposure to a highly proteolytic environment.

Integral OM proteins are generally characterized by long hydrophilic loops and amphipathic transmembrane domains with a hydrophobic exterior and hydrophilic interior. Overall, they tend to exhibit a balanced hydrophobicity profile [29, 30]. However, the hydrophobicity plot of PorN revealed that it was considerably more hydrophilic than OM proteins, inconsistent with localization within the OM but consistent with it being localized to a hydrophilic compartment such as the periplasm (**S1A Fig**). PorK was only slightly more hydrophilic than the Omps tested (**S1A Fig**).

### PorL and PorM form a stable complex

**Fig 6:**
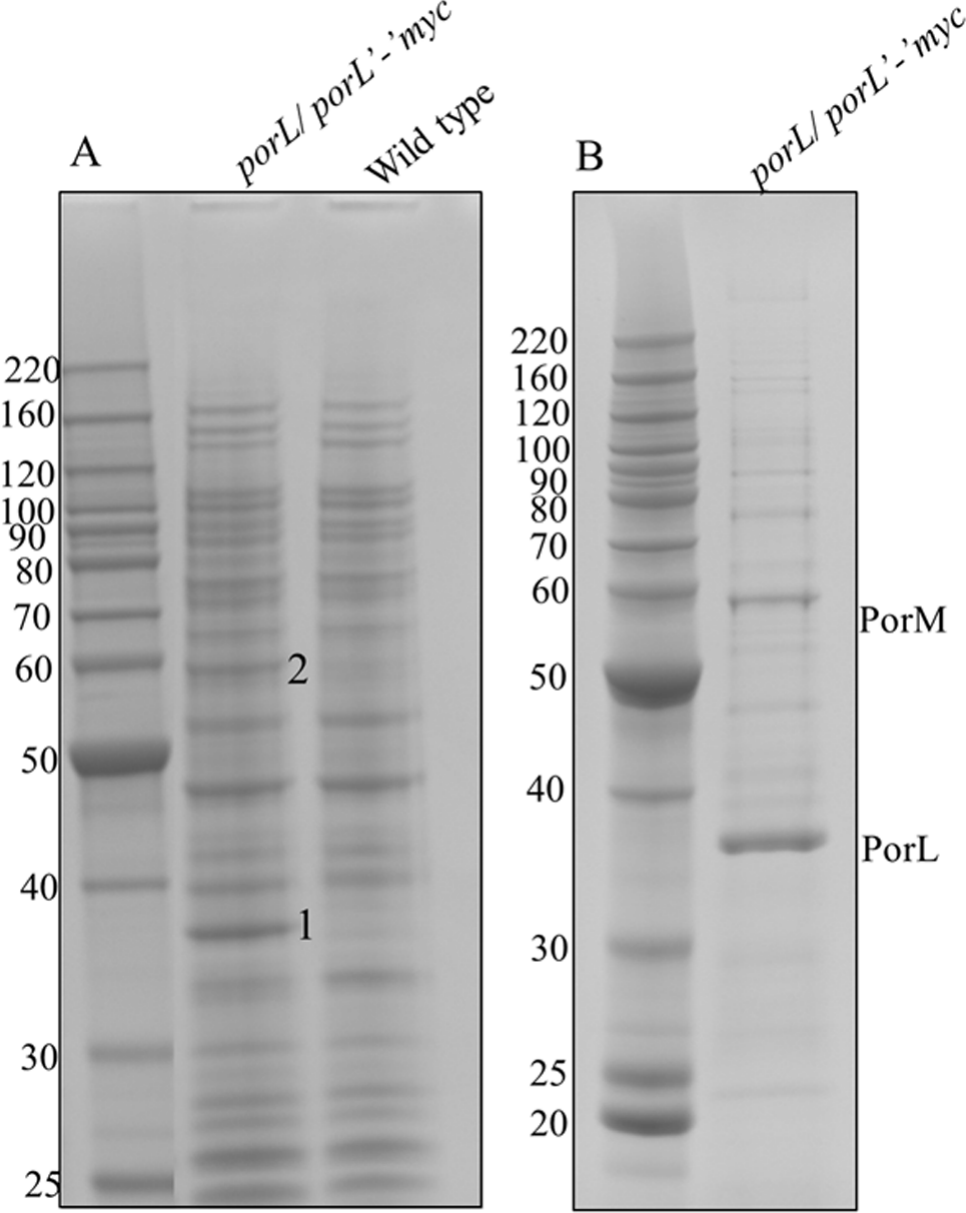
PorL and PorM form a stable protein complex. *porL/porL’-’myc* and wild type *P*. *gingivalis* were lysed in DDM and the protein complex associated with PorL-myc was immunoprecipitated using myc agarose. Bound complexes were eluted with either SDS-loading buffer (A), or with myc peptide (B). The eluted complexes were separated by SDS-PAGE and stained with Coomassie blue. The indicated bands in (A) were identified by MS to be 1- PorL and 2-PorM. All the bands in (B) were also identified by MS (**Table 2, S5 Table**)

Given that the authors of Sato et al. suggested that PorK, PorL, PorM and PorN form a large complex together [8], we assumed that PorL and PorM were perhaps dissociating from the complex during the purification process. To test this hypothesis, we performed co-immunoprecipitation on *porL/porL’-‘myc P*. *gingivalis* strain using Myc agarose. This strain has been characterized previously and shown to have a functional T9SS [8]. Elution of the immunoprecipitated complex with SDS revealed two prominent bands that were only present in the *porL/porL’-‘myc* strain and not in the wild type strain which was used as a negative control (**Fig 6A**). These two bands were identified as 1- PorL and 2- PorM by MS. In addition to this the complex was also eluted using myc peptide (**Fig 6B**) and all the bands were identified by MS (**S5 Table**). The two major bands were identified as PorL and PorM (**Table 2**). Of note, PorK and PorN were not identified, suggesting that PorL and PorM are able to form a stable complex separate from PorK and PorN.

**Table 2:** Identification of PorL and PorM protein bands by mass spectrometry.

Name	Accession No.	Mascot Score	Seq. coverage (%)	No. of peptides
PorL	PG0289	1264	50	10
PorM	PG0290	1146	41	18

### Chemical cross-linking confirms interactions between PorK and PorN and reveals a new putative OM component of the T9SS

**Fig 7:**
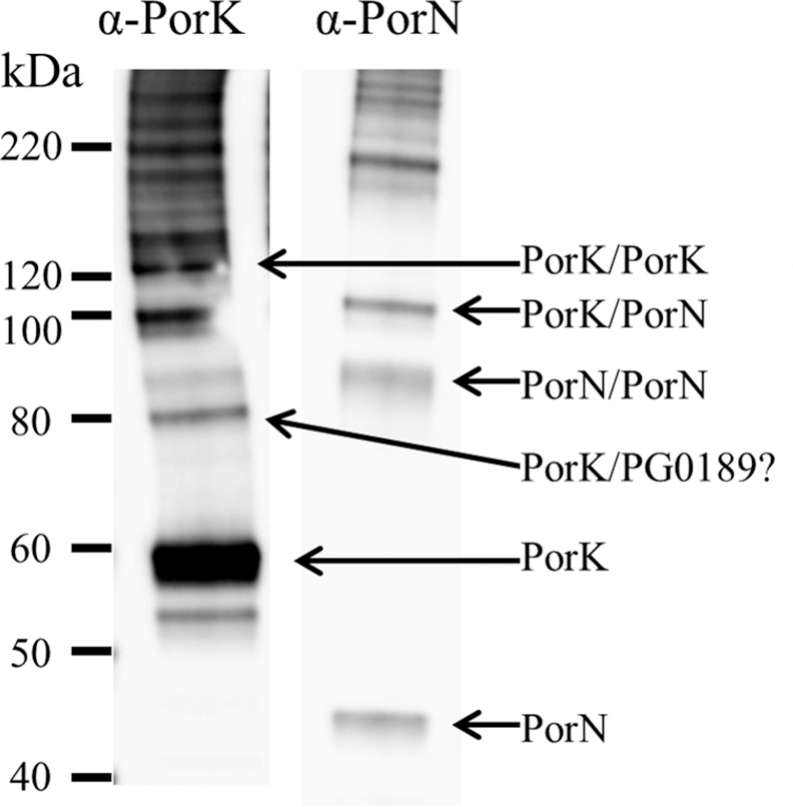
Chemical cross-linking of PorK/N complexes. Purified PorK/N complexes were cross-linked with BS3 for 15 min at room temperature. SDS gel loading dye was added to the samples and boiled for 5 minutes. Proteins in the samples were separated on SDS-PAGE gel, transferred to a nitrocellulose membrane and probed with PorK and PorN antibodies. The PorK and PorN monomers together with putative cross-linked dimers are labeled.

In order to further characterise the interactions within the PorK/N complex, we cross-linked the purified complex with bis(sulfosuccinimidyl) suberate (BS3) and analysed the cross-linked products by western blot. The immunoblot probed with both anti-PorK and anti-PorN exhibited major bands at ~58 kDa and ~45 kDa consistent with the expected PorK and PorN monomers respectively, and higher MW bands consistent with cross-linked products (**Fig 7**). The bands at ~85 kDa (anti-PorN), ~100 kDa (both antisera) and ~120 kDa (anti-PorK) are consistent with PorN-PorN, PorK-PorN and PorK-PorK cross-linked dimers respectively. The ~80 kDa band detected by anti-PorK suggests that PorK may also interact with a ~22 kDa protein.

The cross-linked sample was also digested with trypsin and analysed by LC-MS. To identify cross-linked peptides, the MS data were analysed by pLink software. Beside the many intra-protein cross-links observed **(S6 Table)** seven highly significant inter-protein cross-links were identified involving PorK or PorN confirming the PorN-PorN and PorK-PorN interactions observed by the immunoblot analysis (**Table 3)**. The PorN-PorN cross-link shown in **Table 3** must be inter-protein since the two cross-linked residues are the same residue in each cross-linked protein. Four of the observed cross-links involved a third protein, PG0189, which has a predicted MW of 23 kDa after signal peptide removal. All but one of these cross-links were between PorK and PG0189 (**Table 3**) and this interaction corresponds to the ~80 kDa band detected by anti-PorK (**Fig 7**). An immunoreactive band corresponding to PorN-PG0189 was not observed (**Fig 7**), suggesting that the PorN-PG0189 cross-link observed by LC-MS (**Table 3**) occurs at low frequency. Despite the presence of PG1626, and many other proteins of low abundance in the sample (**Fig 2B**, **S1 Table**), PG0189 was the only protein found to be cross-linked to PorK or PorN (**S6 Table**). Annotated MS spectra for each of the seven inter-protein cross-links are shown (**S2 Fig**) providing detailed evidence for the presence of both peptides in each spectrum.

**Table 3:** Cross-linking and mass spectrometry demonstrate interactions between PorK, PorN and PG0189.

Interacting Proteins^a^	Cross-linked peptide sequences^b^	E-value	Mass error (ppm)
PorN (321)-PorN (321)	G**K**NITSR-G**K**NITSR	1.7E-05	0.7
PorK (342)-PorN (351)	MGDSNN**K**YPWSTEDLR-N**K**AATR	7.9E-05	3.4
PorK (39)- PorN (321)	AVGGELTGA**K**LSSWNEPSPFGMIQVPR-G**K**NITSR	6.1E-04	0.5
PorK (39)-PG0189 (125)	AVGGELTGA**K**LSSWNEPSPFGMIQVPR-SEFNFLPYSDGY**K**YLGTAR	5.4E-10	1.1
PorK (480)-PG0189 (125)	TSIAFSSG**K**APK- SEFNFLPYSDGY**K**YLGTAR	6.2E-05	1.7
PorK (483)-PG0189 (125)	AP**K**SSR- SEFNFLPYSDGY**K**YLGTAR	1.1E-05	1.3
PorN (321)-PG0189 (125)	G**K**NITSR-SEFNFLPYSDGY**K**YLGTAR	1.3E-09	0.0

^a^ The number in parentheses indicates the position of the cross-linked Lys residue within the protein sequence

^b^ The cross-linked Lys residues are shown in bold and underlined

### PG0189 is predicted to be an outer membrane component of the T9SS

All known components of the T9SS are conserved amongst other species that contain the T9SS but are absent in the closely related species *Bacteroides fragilis* and *Bacteroides thetaiotaomicron* [7, 8]. A BLAST search of PG0189 using BLASTP did not return a sufficient number of hits to assess its species distribution however two iterations of PSI-BLAST demonstrated that the homologs of PG0189 exhibit a species distribution consistent with it being a component of the T9SS **(S7 Table)**. In a previous study [31], PG0189 was localised to the OM of *P*. *gingivalis* and was predicted to be an OM β-barrel protein by both Pfam and the Transmembrane β-barrel database. Structural modelling of PG0189 using Phyre-2 resulted in strong hits to 8-stranded outer membrane β-barrel proteins including the β-barrel domain of OmpA from *E*. *coli* and its homologs in *Klebsiella pneumoniae* and *Pseudomonas aeruginosa* (OprF), and also to full length OmpW from *E*. *coli* and its homolog OprG in *Pseudomonas aeruginosa* **(S3 Fig)**. For each of the best models, the region of PG0189 cross-linked to PorK and PorN was located in a putative periplasmic loop consistent with the periplasmic localisation determined for PorK and PorN in this study **(S3 Fig)**.

### PorN is essential for the stable expression of PorK, PorL and PorM

**Fig 8:**
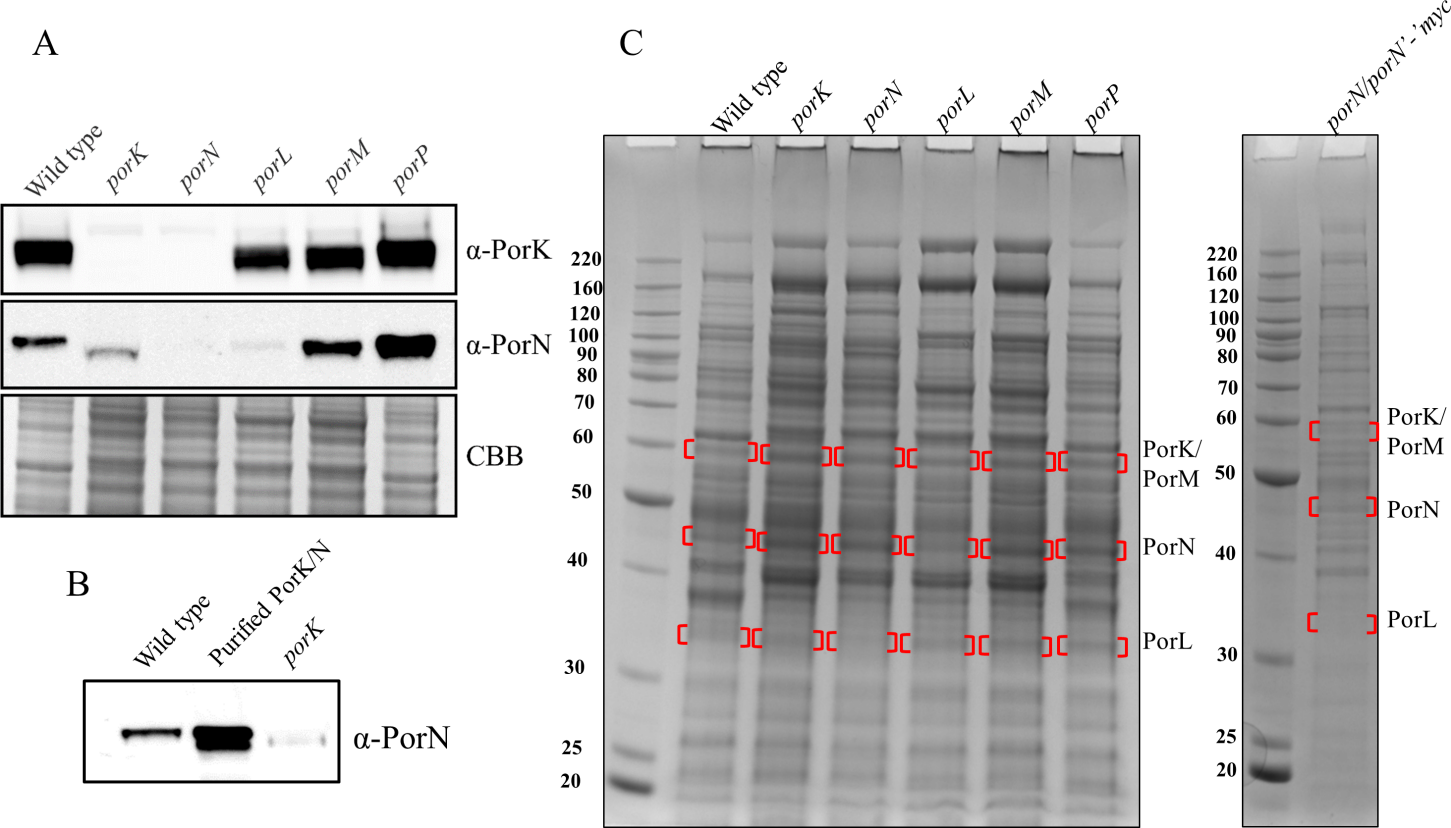
PorN required for the stable expression of PorK, PorL and PorM. (A) Whole cell lysates from wild type, *porK*, *porN*, *porL*, *porM* and *porP* mutants were separated on SDS-PAGE and transferred onto a membrane. The membrane was probed with PorK and PorN antibodies. Coomassie blue (CBB) stained gel shows the relative loading amount. (B) Whole cell lysates from wild type, *porK* mutant and purified PorK/N complexes were immunoblotted using PorN antibodies. (C) Whole cell lysates from wild type, *porK*, *porN*, *porL*, *porM*, *porP* and *porN/porN’-’myc* were separated on SDS-PAGE and stained with Coomassie blue. The bands indicated by red brackets were excised, subjected to in gel trypsin digestion and identified by mass spectrometry (**Table 4**).

To further explore the interactions between these proteins and their functions we analysed the respective mutants using immunoblots and mass spectrometry. Whole cell lysates from wild type, *porK*, *porN*, *porL*, *porM* and *porP* mutant *P*. *gingivalis* cells were subjected to SDS-PAGE and immunoblot analysis. The blot was probed with anti-PorK and anti-PorN antibodies. PorK was present in all mutants except for *porK* and *porN* mutants (**Fig 8A**). The absence of PorK in the *porK* mutant is expected as the *porK* gene has been deleted, however, the absence of PorK in the *porN* mutant indicates that PorK is not stable in the absence of PorN and perhaps is degraded. Immunoblot with anti-PorN antibodies revealed markedly reduced amounts of PorN in the *porL* mutant and interestingly PorN in the *porK* mutant migrated to a lower apparent molecular weight than the wild type and the other mutants (**Fig 8A**). Purified PorK and PorN complex separated on SDS-PAGE produced two bands corresponding to PorN (**Fig 2B**). The migration of these two bands was directly compared to the PorN bands in whole cell lysates obtained from the wild type and *porK* mutant strains **(Fig 8B**). The higher band in the purified complex corresponded to the intact native form of PorN while the lower band appeared to be a degraded form. The form present in the *porK* mutant also appeared to be the degraded form. Together, this suggests that PorL is crucial for the stable expression of PorN at high levels while PorK may protect a portion of PorN from degradation. To analyze the expression of PorL and PorM in these mutants, whole cell lysates were generated for each strain and subjected to SDS-PAGE. Bands were excised according to the known migration of these proteins (shown by red brackets in **Fig 8C**) and the proteins present were identified by mass spectrometry. While many proteins were identified in each band, Table 4 shows the identification of the proteins of interest (PorK, PorL, PorM and PorN). Surprisingly in the *porN* mutant, PorL, PorM and PorK were not identified while the other mutants were only lacking the protein corresponding to the deleted gene. To verify that the *porN* mutant was not compromised, the *porN* complemented strain with *porN’-‘myc* was analyzed. All three, PorK, PorL and PorM, were restored in the complemented strain (**Table 4**). Collectively, these results suggest that PorN plays a central role in the assembly or stabilization of these T9SS components.

**Table 4:** Identification of PorK, PorN, PorL and PorM proteins from the mutant strains by mass spectrometry (see Fig 8C). The mascot scores obtained are shown.

Mutants	Wild type	*porK*	*porN*	*porL*	*porM*	*porP*	*porN/porN’-‘myc*
PorK	1115	-	-	521	643	979	1202
PorN	815	555	-	224	520	638	444
PorL	916	657	-	-	418	853	1155
PorM	1674	1233	-	347	-	1939	1581

### Formation of the ring structure requires both PorK and PorN and the ring structure is not affected by the absence of PorL and PorM

**Fig 9:**
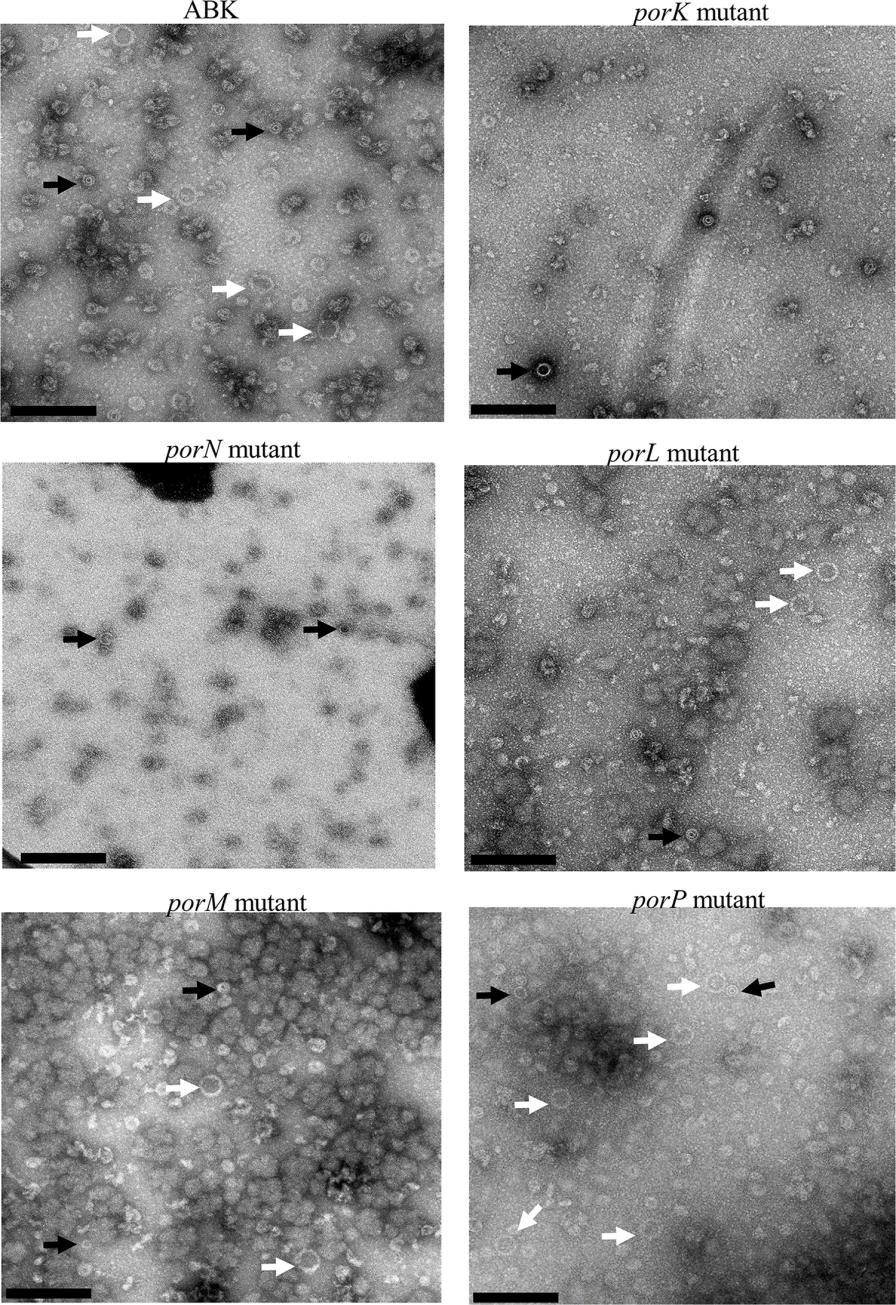
Electron micrographs showing presence of large (50 nm) rings in *porL*, *porM* and *porP* mutants but not in *porK* and *porN* mutants. Preparations of large protein complexes were isolated from wild type lacking gingipains (33277ABK), *porK*, *porN*, *porL*, *porM* and *porP* mutants. The samples were negatively stained and viewed under the electron microscope. White arrows indicate ring structures that are consistent with the PorK/N complex. Black arrows indicate a smaller ring (~ 30 nm) that was distinct from the PorK/N complex and present in all samples. Scale bars, 200 nm.

Since the expression levels of PorK, PorL, PorM and PorN were altered in different mutants we investigated whether the formation of the ring structure was affected in any of these mutants and whether PorN could form a ring structure in the absence of PorK. A purification for macromolecular structures was performed on wild type lacking gingipains (33277ABK), *porK*, *porL*, *porM*, *porN* and *porP* mutant strains using the sucrose cushion method. Samples from each strain were negatively stained and visualized under the electron microscope. PorK/N rings (50 nm in size) were observed in all strains except for the *porK* and *porN* mutants (marked with white arrows) (**Fig 9**). Together this indicates that both PorK and PorN are required for the formation of the ring structure and the structure is not affected by the absence of PorL, PorM and PorP.

## Discussion

The type IX secretion system (T9SS) is a newly discovered secretion system that is specific to the *Bacteroidetes* phylum [7, 8, 24]. Numerous components of the T9SS have been identified, however, the precise role of many of these components remain a mystery. PorK, PorL, PorM and PorN have been shown to be components of the T9SS as the mutants lacking these proteins have no cell surface gingipain activity and the CTD proteins accumulate in the periplasm [8]. Further to this we have now shown that *porK*, *porL*, *porM* and *porN* mutants lack an EDSL, which is also characteristic of *P*. *gingivalis* mutants with a defective T9SS [22, 26]. Here we show for the first time that PorK and PorN form a very large ring-shaped structure with an outside diameter of 50 nm and an internal diameter of 35 nm. This is the first report on the structure of any protein complex of the T9SS. The complex appears to have a 32-fold to 36-fold symmetry and the molar ratio for PorK:PorN was close to 1:1. Based on the subunit composition and molar ratio, the calculated molecular size of the DDM complexes (2x PorK rings, 2x PorN rings, Fig 4E *in-vitro*) would be between 5.9 MDa (32- fold) and 6.6 MDa (36- fold) which is consistent with the molecular weight obtained by blue native PAGE analysis (≥ 4.5 MDa) since the estimated mass was based on a standard protein band which is greater than or equal to 10 MDa [32]. The MW of the proposed *in-vivo* complex (1x PorK ring, 1x PorN ring, Fig 4E) would therefore be around 3 MDa. Cryo-electron microscopy revealed that each ring was composed of two sub-rings, with one sub-ring being larger than the other. Although our studies did not allow us to assign a particular protein to each component of the ring, we propose that the larger sub-ring is composed of PorK subunits and the smaller sub-ring is composed of PorN subunits. This is based on (i) PorK having a higher molecular weight than PorN and hence may form a larger ring than PorN given the same number of subunits; (ii) The rings were bound to each other via the larger sub-rings perhaps due to hydrophobic interactions caused by the lipid portion of PorK (Fig 4E, *in-vitro*). Again this is suggestive of the larger sub-ring being PorK.

The study by Sato et al., suggested that PorK, PorL, PorM and PorN form a large complex using blue native analysis [8]. However, it was unclear whether these proteins were represented by one or more complexes. Recently, in a separate blue native PAGE study of *P*. *gingivalis* membrane preparations, a large complex was observed and found to contain PorK and PorN, but not PorL or PorM, suggesting that PorK and PorN form an independent stable complex [33]. In the current study, this interpretation was confirmed as PorL and PorM did not co-purify with the PorK/N complex and also immunoprecipitation studies with PorL-myc identified PorM to be the only stable interacting partner of PorL. It is possible that all four proteins interact *in vivo*, with the interactions between PorK/N with PorL/M being less stable. Core structures spanning both the inner and outer membranes have been isolated for Type III and Type IV secretion systems, which are both one-step secretion systems that secrete proteins directly from the cytoplasm to the exterior [34]. The Type II secretion system (T2SS) secretes folded proteins from the periplasm into the extracellular environment and it consists of four main parts: the outer membrane complex, a periplasmic pseudopilus, an inner membrane platform and a cytoplasmic ATPase [34, 35]. However due to the interactions being very dynamic a core structure containing both inner and outer membrane components has not yet been isolated [35]. The T9SS is similar to the T2SS in the sense that it is a two-step system. The CTD proteins that pass through the T9SS have an N-terminal signal sequence that allows them to translocate through the IM via the SEC complex and the C-terminal signal is then required for exit through the OM via the T9SS. Like other systems the T9SS may be dynamic in nature making a fully assembled system difficult to isolate.

Previously it was shown that PorK and PorN are localized to the outer membrane based on differential membrane solubility in Triton X-100 [8]. However, it is unknown whether they are integral or peripheral membrane proteins and if peripheral whether they are located on the cell surface or on the periplasmic side of the OM. In this study, we show that PorK and PorN were digested with proteinase K rapidly after lysis compared with that using intact *P*. *gingivalis*. LptO and HmuY, which are known integral and peripheral (cell surface) outer membrane proteins, respectively, were not readily digested with proteinase K in either lysed or intact *P*. *gingivalis*. This suggested that PorK and PorN are localized in the periplasm and are protected from exogenous proteinase K, but not inherently resistant to proteinase K. Further confirmation of this localization was obtained via cross-linking. Both PorK and PorN were cross-linked to a putative periplasmic loop of the outer membrane protein PG0189. Collectively, these data indicate that the PorK/N complex is localized in the periplasm and anchored to the outer membrane via the PorK lipoprotein.

To our knowledge, there are two other large ring-shaped structures that are located in the periplasm of other Gram- negative bacteria. First is the flagellar P-ring (FlgI) that assembles around the flagellar rod and is thought to be attached to the peptidoglycan layer [36, 37]. It forms a 20 nm ring composed of 26 subunits of FlgI protein and it is a non-rotating component that functions to hold the central rod in place [38]. The P-ring also directs the assembly of the L-ring (FlgH) which forms a pore in the outer membrane required for hook polymerization [39]. Second is the VirB9 protein of the type IV secretion system that forms a ring around VirB10 in the periplasm and is stabilized by VirB7 [40]. The diameter of the VirB9 ring is 18.5 nm and is composed of 14 subunits of VirB9 [4]. Its exact role in the function of the T4SS is not known, however it is absolutely necessary for the formation of the T4SS outer membrane complex which consists of VirB7, VirB9 and VirB10 and which together form a channel through the OM and part of the periplasm [40]. VirB9 has also been shown to interact with the effector protein substrates [41]. In both cases, the known periplasmic ring structure (FlgI and VirB9) interact with other proteins internal to the rings suggesting that this is also likely for the PorK/N ring. In comparison to the two rings mentioned above, the PorK/N rings are much larger (50 nm) suggesting that whatever proteins are internal to the ring also form a large diameter structure. One or more of these proteins may form the OM pore structure which may extend into the periplasm and interact with the inside of the ring. Presumably, the OM pore would be much smaller than the internal diameter of the PorK/N ring (35 nm) as known OM pores range from 1.6–15.5 nm [34]. Nevertheless, a large opening would be consistent with the prolific secretion of large folded substrates such as the gingipain precursors (180 kDa) and other large substrates such as the “CHU large proteins” (up to 297 kDa) in other *Bacteriodetes* [7]. PG0189 is one candidate for the OM pore structure. However, the small size (23 kDa) and relatively low amounts of PG0189 found associated with PorK and PorN suggest that there may be insufficient protein to oligomerise to form a pore hence it may have a different function perhaps in assembly and/or stability.

We also identified that PorN was required for the stable expression of PorK, PorL and PorM. Moreover, we observed that in the *porK* mutant, PorN migrates lower on SDS-PAGE suggesting that some regions of PorN are protected from proteolysis by interaction with PorK. Furthermore, it was recognized that PorN on its own does not form a stable ring-shaped structure and the PorK/N ring structure was not affected by the absence of PorL and PorM. The secretion systems in Gram- negative bacteria are assembled in a stepwise manner, for instance, in T2SS the inner membrane complex (GspEFLM) assembles first followed by peptidoglycan remodeling and secretin oligomerization in the OM [42]. Similarly, in the T4SS, the assembly begins with the formation of the core complex consisting of VirB7, 9 and 10 at the outer membrane followed by the formation of the inner membrane complex and the pilus [40]. PorN appears to have a crucial role in assembly of the T9SS as in the *porN* mutant, PorK, PorL and PorM were absent and all four proteins were restored in the PorN complementation strain. However, PorK is also required to protect PorN and enable it to form a stable ring.

**Fig 10:**
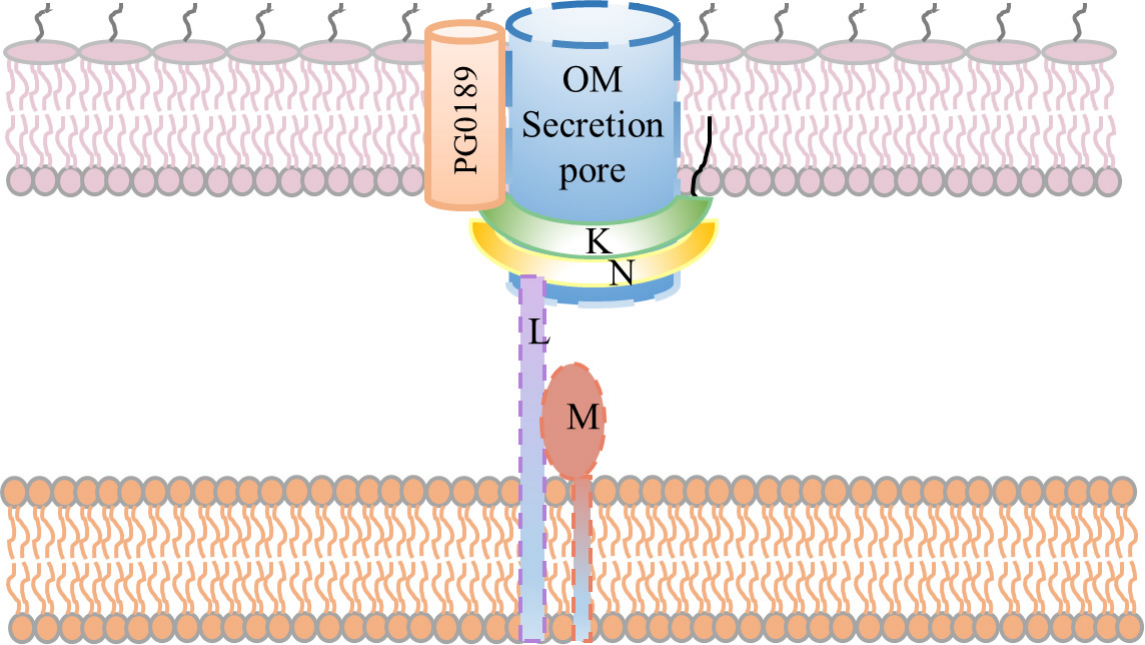
A proposed model for the roles and interactions of PorK, PorL, PorM, PorN and PG0189 in the T9SS. PorK and PorN interact to form a ring-shaped structure that is localised in the periplasm and tethered to the outer membrane via the PorK lipid moieties (lipid shown as black line). This structure may be further stabilised by its association with the PG0189 outer membrane protein. It is proposed that the PorK and PorN rings assemble around the periplasmic extensions of the unknown OM secretion pore. Both PorL and PorM have transmembrane spanning domains and are proposed to transduce energy from the inner membrane (PMF) or cytosol (ATP) and power secretion of the T9SS substrates through the transient interactions with the PorK/N complex. The topology of the PorL and PorM inner membrane proteins is not known.

With the current knowledge of the T9SS and the structure we have obtained of PorK/N in this study we suggest the following model for the broad roles of the PorK/N and PorL/M complexes within the T9SS (**Fig 10**). As shown in the diagram, the PorK/N ring complex is localized in the periplasm and is associated with the OM via the PorK lipid. We speculate that the ring complex is stabilised by its association with the PG0189 outer membrane protein, and that it assembles around the periplasmic extensions of the outer membrane pore protein(s), which is yet to be identified. All secretion systems require ATP or PMF to power the secretion of the protein or DNA substrates. It is unclear in *P*. *gingivalis* whether ATP or the PMF is utilized as the source of energy. It has been shown that PMF powers gliding motility in *Flavobacteria johnosoniae*, which is linked to the T9SS [43] and therefore this is also likely to be the case for *P*. *gingivalis*. Inner membrane components are required to transduce the energy, to date the only two known T9SS components that are localized in the inner membrane are PorL and PorM, therefore we propose PorL/M to be involved in transducing the cellular energy to power secretion through dynamic interactions with PorK/N (**Fig 10**). A similar role has been suggested for GldL and GldM which are orthologs of PorL and PorM, respectively in *F*. *johnosoniae* [24, 43, 44].

In conclusion, we present for the first time the structure of part of the T9SS, the PorK/N ring complex, which by nature of its size and shape is likely to be part of the translocation machinery. Further studies involving electron cryo-tomography of the complex and crystal structures of the PorK and PorN subunits will enable a higher resolution structure to be determined. Furthermore, identification of the outer membrane secretion pore protein and its structural relationship with the PorK/N ring complex will greatly enhance our understanding of the T9SS.

## Materials and Methods

### Bacterial strains and culture conditions

*P*. *gingivalis* strains W50 and ATCC 33277 were grown in tryptic soy enriched Brain Heart Infusion broth (TSBHI) (25 g/L Tryptic soy, 30 g/L BHI) supplemented with 0.5 mg/mL cysteine, 5 μg/mL haemin and 5 μg/mL Menadione. For blood agar plates, 5% defibrinated horse blood (Equicell, Bayles, Australia) was added to enriched trypticase soy agar. Mutant strains were grown in the same media as above with the appropriate antibiotic selections. All *P*. *gingivalis* strains were grown anaerobically (80% N_2_, 10% H_2_ and 10% CO_2_) at 37°C. *porK*, *porL*, *porM*, *porN*, *porP*, *porL/porL’-‘myc*, *porN/porN’-‘myc* ATCC 33277 *P*. *gingivalis* mutant cells and ABK mutant lacking gingipains have been characterized previously [8, 45]. The W50 *kgp* ^*-*^ *rgpA* ^*-*^ *rgpB* ^*-*^ triple mutant that had spontaneously developed a mutation in the *wbaP* gene (W50ABK*WbaP) and therefore lacks A-LPS has been described previously [9, 46].

### Purification of PorK and PorN complexes

At first, the PorK and PorN complex isolation was carried out according to a T3SS needle complex purification method described previously [3], and then with the following modified method. Briefly, washed *P*. *gingivalis* W50ABK*WbaP mutant cells were resuspended in lysis buffer (50 mM Tris, pH 7.5, 150 mM NaCl. 0.5 M sucrose, 5 mM MgCl_2_, 1% n-Dodecyl β-D-maltoside (DDM) (Sigma-Aldrich), complete protease inhibitor cocktail (Roche) and benzonase (Sigma-Aldrich)) and incubated in ice for 45 min. Unbroken cell debris was removed by centrifugation at 10000 g for 25 min at 4°C. The supernatant was centrifuged at 142000 g for 40 min at 12°C and the pellet was resuspended in 50 mM Tris pH 7.5, 500 mM NaCl, 1% DDM, 5 mM EDTA, 0.5 mg/ml lysozyme and complete protease inhibitor cocktail. The sample was incubated at 37°C for 15 min and centrifuged again at 142000 g for 40 min. The pellet was resuspended in 1% DDM, 10 mM Tris, 500 mM NaCl and 5 mM EDTA and loaded onto a final concentration of 30% w/v CsCl. The sample was centrifuged at 214200 g for 17 hours in a Beckman TLS-55 rotor. Eight fractions were collected and combined in a buffer containing 0.5% DDM, 10 mM Tris, 500 mM NaCl and 5 mM EDTA. The sample was further centrifuged at 543200 g for 30 min in a MLA-130 rotor. The pellet containing complexes was resuspended in 0.5% DDM, 10 mM Tris and 500 mM NaCl and stored at 4°C. Single rings were obtained by resuspending the pellet in 0.3% lauryldimethyl oxide (LDAO) instead of DDM. For isolation of complexes from wildtype W50 *P*. *gingivalis*, LDAO was used instead of DDM.

A crude preparation of PorK and PorN complex was performed as follows. The *P*. *gingivalis* cells were lysed and digested with lysozyme as above and the pelleted complexes were resuspended in 50 mM Tris-HCL, pH 7.4, 500 mM NaCl, 1% DDM and 5 mM EDTA. Following centrifugation at 17,000 g for 10 min, the resulting supernatant was layered on a 30% sucrose cushion and centrifuged at 35,000 rpm for 2 hours in a TLS-55 rotor (Beckman Coulter). The pellet was resuspended in 10 mM Tris-HCl, pH 7.5, 50 mM NaCl and 0.5% DDM and stored at 4°C.

### SDS-PAGE and mass spectrometry

An aliquot of sample was mixed with SDS- loading dye and analysed on NuPAGE Bis-Tris precast gels. The gels were electrophoresed using 3-(N-morpholino) propane sulfonic acid (MOPS) SDS running buffer (Life technologies). The gels were stained overnight with 0.1% w/v colloidal Coomassie Blue G-250. Protein gel bands were excised and washed in 50 mM NH_4_HCO_3_/ethanol 1:1 solution, and reduced and alkylated with dithiothreitol (DTT) and iodoacetamide respectively. In gel tryptic digestion was performed overnight at 37°C using sequencing grade modified trypsin (10 ng/μl) (Promega, NSW, Australia). The resulting peptides were analysed with either LC-MS/MS system comprising an Ultimate 3000 HPLC (Dionex) and an Esquire HCT Ultra ion trap [31] or with LTQ Orbitrap Elite mass spectrometer with a nano ESI source interfaced with an ultimate 3000 RSLC nano-HPLC [9]. Proteins were identified via Mascot MS/MS ions search using the following settings: protein database = *P*. *gingivalis* W83 or *P*. *gingivalis* ATCC33277 as appropriate, enzyme = trypsin, fixed modification = carbamidomethyl (Cys), variable modification = oxidation (Met), missed cleavages = 1, MS tolerance = 1.5 Da (ion trap), 10 ppm (orbitrap), MS/MS tolerance = 0.5 Da (ion trap), 0.2 Da (orbitrap). For quantitation, MaxQuant software (v1.5) was used with label free quantitation (using the LFQ metric) with the same database and modifications as above, and all other parameters set at default.

### Electron microscopy

Purified complexes were adsorbed onto glow discharged Formvar-carbon films supported on 200 mesh copper grids. Samples were negatively stained with aqueous uranyl acetate and viewed under an FEI Tecnai F30 microscope at the Bio21 Institute Advanced Microcopy Facility. For tomography, negatively stained samples were tilted from -65 to +65 degrees and micrographs were collected at every 2 degrees tilt. Tilt series alignment and tomogram reconstruction was performed using IMOD [47]. For Cryo-electron microscopy, samples from either purified complexes or washed *P*. *gingivalis* cells were applied onto a glow discharged Quantifoil R3.5/1 holey carbon film mounted on a 200 mesh copper grid. Excess liquid was removed by blotting with double layered filtered paper and the grid was plunge frozen in liquid ethane. Transmission electron microscopy was carried out under cryogenic conditions using a FEI Tecnai F30 that was operated at 200 or 300 kV and was equipped with a Gatan US1000 2kX2k CCD camera. Micrographs were obtained under low-dose conditions (~1000 e^-^/nm) with underfocus values of 4–6 μm. Single particle analysis was performed on 107 particles with a symmetry of d36 using EMAN2 [48].

### Proteinase K susceptibility and immunoblot analysis

W50ABK*WbaP *P*. *gingivalis* cells were grown to an O.D of 0.8. The cells were washed with phosphate buffered saline (PBS) once and were resuspended in the same buffer. The cells were divided into two tubes, the cells in one tube were processed by French press treatment (pressure set at 200 psi) and the cells in the second tube were left untreated. Proteinase K (100 μg/ml) was added to all tubes and an aliquot was collected at 0 (10 s), 1, 4 hr and overnight. The samples from all aliquots were lysed in SDS loading dye and resolved by SDS-PAGE. The proteins were then transferred to a nitrocellulose membrane (100V for 1 hour) and probed with the following antibodies: anti-PorK and anti-PorN [8], anti-HmuY (a kind gift from Professor Olczak) [28] and anti-LptO [22] which also contained anti-GroEL and anti-translational elongation factor Tu (anti-EFtu) antibodies (**S4 Fig**).

### High sensitivity amino acid analysis

Purified PorK/N complexes were separated on SDS-PAGE and transferred to PVDF membrane using CAPS transfer buffer (pH 11.0) at 60V for 2 hours. The membrane was then soaked in 0.1% Coomassie R-250 for one minute and destained with 50% methanol. The bands corresponding to PorK and PorN were excised and transferred into a tube. The samples were then sent to the Australian proteome analysis facility (APAF, www.proteome.org.au) for high sensitivity amino acid analysis.

### Co-immunoprecipitation

*P*. *gingivalis porL/porL’-‘myc* were lysed in 50 mM Tris-HCl, pH 7.5, 150 mM NaCl. 0.5 M sucrose, 5 mM MgCl_2_, 1% DDM, 1x complete protease inhibitor cocktail (Roche), benzonase, 250 units (Sigma-Aldrich) and 5 mM Nα-tosyl-l-lysine chloromethyl ketone hydrochloride (TLCK, Sigma-Aldrich) and incubated on ice for 45 min. Unlysed cells and debris were removed by centrifugation at 10000 g for 25 min at 4°C. The lysate was then incubated with Myc agarose (MBL International) for 1 hour at 4°C with rotation. The agarose was then treated with 0.5 mg/ml lysozyme in 50 mM Tris pH 7.5, 500 mM NaCl, 1% DDM, 5 mM EDTA, and incubated at 37°C for 15 min followed by washes in 50 mM Tris-HCl, 150 mM NaCl and 0.1% DDM. The agarose was then either resuspended in SDS loading buffer and heated at 95°C for 10 min or was incubated with 40 μL of myc peptide (1 mg/mL for 10 min on ice to elute the complexes bound to the Myc agarose). The eluted complex was then separated by SDS-PAGE and the bands were identified by mass spectrometry.

### Blue Native PAGE

Purified PorK/N complexes were resuspended in 10 mM Tris-HCl, pH 7.5, 50 mM NaCl, 10% glycerol and 0.5% DDM. Native Page G-250 sample additive (Life Technologies) was added to the resuspended sample to a final concentration of 0.125% before loading a NativePAGE 3–12% Bis-Tris gel. The gel was subjected to electrophoresis at 150 V for 1 hour at 4°C then the voltage was increased to 250 V until the dye front reached the bottom of the gel. Supercomplexes, pyruvate dehydrogenase complex of ≥ 10000 kDa and the tetrametric form of ATP synthase of 2400 kDa [32] were obtained from bovine heart mitochondria (Abcam), solubilized in 1% digitonin and used as molecular weight standards.

### Cross-linking with BS3 and subsequent MS analysis

PorK/N complexes were purified as described above except that PBS was used instead of Tris before the final centrifugation step to pellet the PorK/N complex. The pelleted complex (~12 μg) was resuspended in PBS containing 0.5% DDM to give a concentration of approximately 0.3 mg/mL of complex. BS3 crosslinker (1 mM) was added to the complex and the mixture was incubated for 15 min or 60 min at room temperature. The reaction was quenched with 20 mM Tris-HCl, pH 7.5. Solid urea was added to the mixture to obtain a final concentration of 8 M urea. The sample was then reduced with 10 mM DTT for 1 h at 37°C followed by alkylation with 25 mM iodoacetamide for 30 min at room temperature in the dark. The sample was then diluted with 25 mM NH_4_HCO_3_ to a final urea concentration of 1 M and digested with 10 ng/μL sequencing grade modified trypsin at 37°C overnight. Digested peptides were desalted and concentrated using Millipore μC18 Zip-tips (Sigma-Aldrich, Sydney, Australia) according to manufacturer’s instructions and the eluted samples were analysed using Orbitrap LC-MS as above, except that samples were analysed with and without the exclusion of 2+ ions.

The MS data was first searched using Mascot (as above) and setting BS3 as an additional variable modification. The sequences of the top 29 proteins identified were used to construct a sequence database for the cross-link search. The cross-link search was performed using pLink v1.23 downloaded from http://pfind.ict.ac.cn/software/pLink/ [49, 50], (with the following settings. Enzyme = Trypsin, missed cleavages = 3, MS tolerance = 5 ppm, cross-linker = BS3, spectra format = mgf, fixed modification = carbamidomethyl_C, variable modification = oxidation_M, spectra type = HCD. Spectral annotation was performed with the aid of pLabel (part of pLink).

### Molecular modelling

The predicted mature PG0189 sequence starting at Gln 29 was modelled using Phyre-2 software at www.sbg.bio.ic.ac.uk/phyre2 using the intensive mode [51]. The models were rotated, formatted and labeled using the UCSF CHIMERA program [52].

## Supporting Information

S1 Fig Bioinformatics analysis of PorK and PorN. (PDF)

S2 Fig Annotated MS/MS spectra for cross-linked peptides. (PDF)

S3 Fig Molecular modelling of PG0189 using Phyre-2. (PDF)

S4 Fig Anti-rLptO antisera also detects GroEL and EFtu. (PDF)

S1 Table MS data for the identification of proteins present in purified PorK/N complex. (XLSX)

S2 Table MS data for blue native PAGE band of purified PorK/N complex. (XLSX)

S3 Table High sensitivity amino acid analysis of PorK and PorN in PorK/N complexes. (DOCX)

S4 Table Quantification of proteins present in PorK and PorN bands using MaxQuant software. (XLSX)

S5 Table MS data for proteins co-immunoprecipitated with PorL and eluted with myc peptide. (XLSX)

S6 Table MS cross-linking data from purified PorK/N complex. (XLSX)

S7 Table Species distribution of PG0189 homologs. (DOCX)
