# Supplementary material for: Structural Insights into the PorK and PorN Components of the Porphyromonas gingivalis Type IX Secretion System
Source: PLoS Pathog. 2016 Aug 10;12(8):e1005820. doi: 10.1371/journal.ppat.1005820 (PMC4980022; doi:10.1371/journal.ppat.1005820)
Supplement: S1 Fig — (PDF) [file ppat.1005820.s001.pdf]

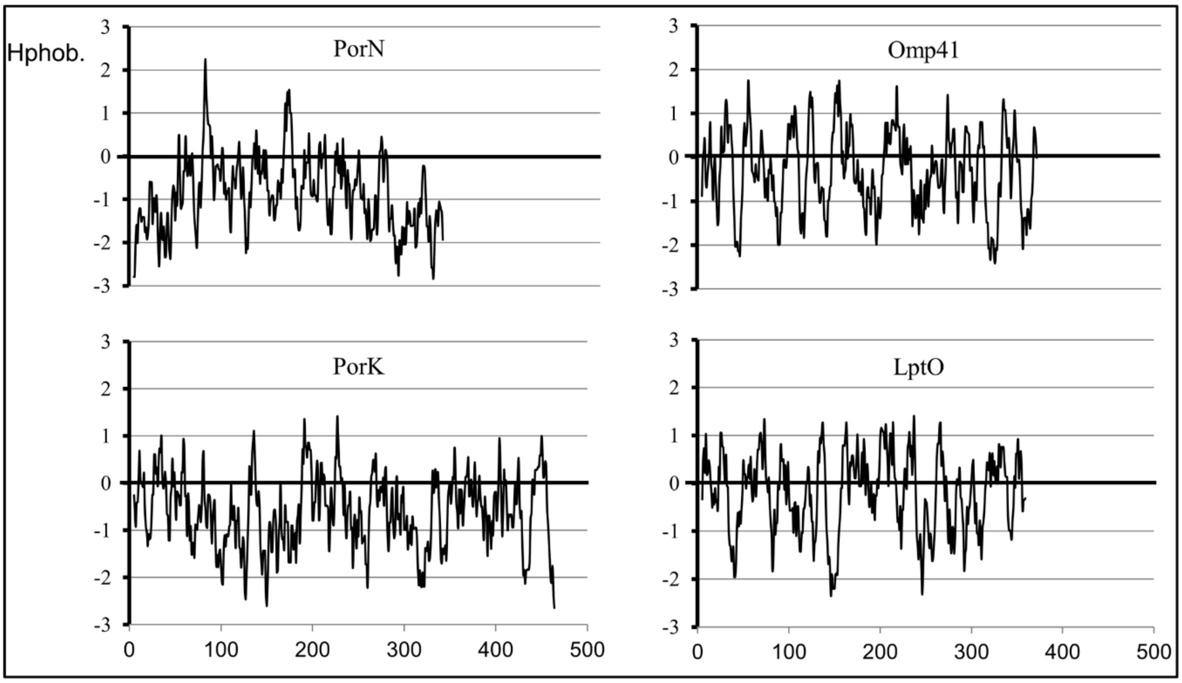

S1 Figure. Hydrophobicity plots of PorK and PorN

(A) Hydrophobicity plots were plotted using an online software http://web.expasy.org/cgi-bin/protscale/protscale.pl PorK and PorN are more hydrophilic in comparison to known outer membrane proteins Omp41 and LptO. While only the OM proteins, LptO and Omp41 are shown, many others were tested giving very similar overall hydrophobicity profiles.
