# Supplementary material for: Structural Insights into the PorK and PorN Components of the Porphyromonas gingivalis Type IX Secretion System
Source: PLoS Pathog. 2016 Aug 10;12(8):e1005820. doi: 10.1371/journal.ppat.1005820 (PMC4980022; doi:10.1371/journal.ppat.1005820)
Supplement: S2 Fig — (PDF) [file ppat.1005820.s002.pdf]

S2 Figure. Annotated MS/MS spectra for cross-linked peptides

Annotated MS/MS spectra are provided for each cross-linked peptide shown in Table 3, and in the same order. In each spectra, the major ions were found to be the y-ions (C-terminal fragments cleaved across the peptide bond). The mass and label for each of these ions is shown. Other ions such as b ions and occasionally a ions and immonium ions are just labeled with their designation (and not their mass). The sequence of the two cross-linked peptides is shown together with the observed fragmentation. The MS/MS ions observed in the spectrum for the first sequence are annotated in orange text while ions corresponding to the second sequence (if different to the first) are annotated in blue text. Note all observed ions are annotated due to space constraints. The charge state (usually 3+ or 4+) is also provided. Note that where ions include the cross-linked lysine residue (e.g spectrum #6, y7-y13), the mass of the entire other peptide and cross-link is present.

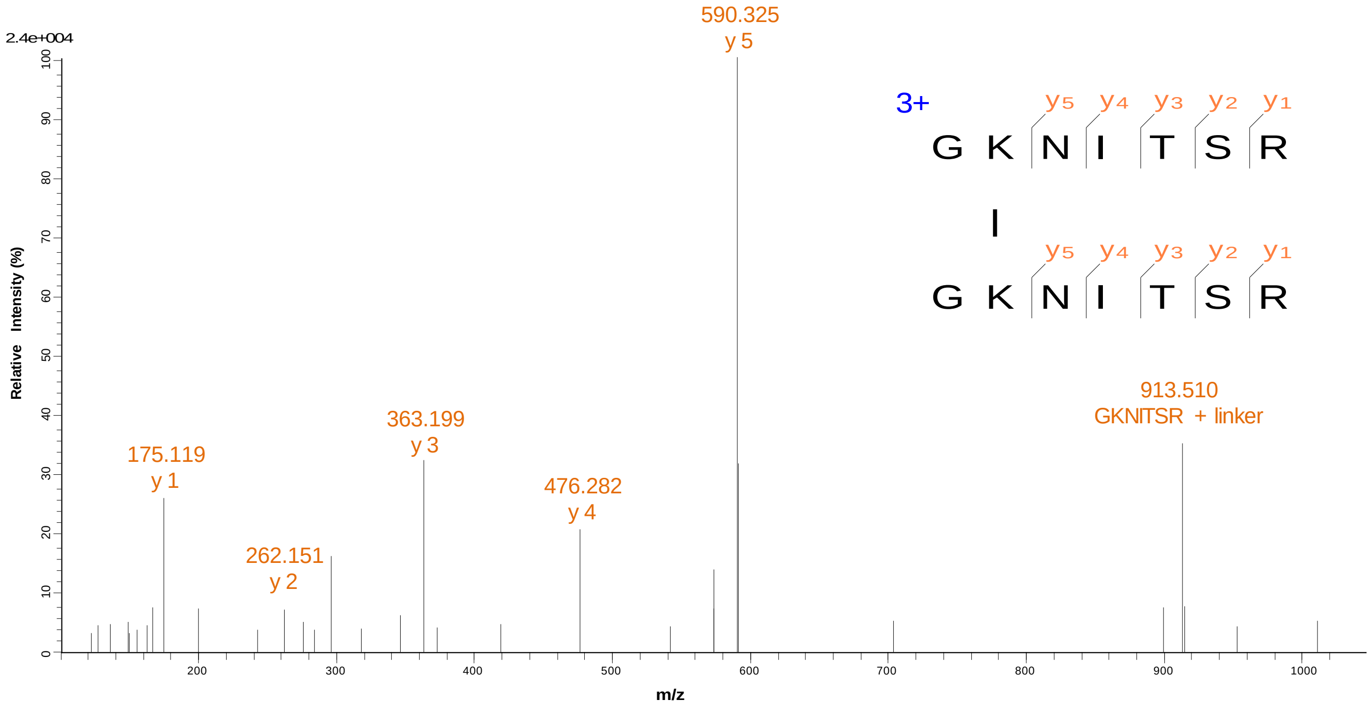

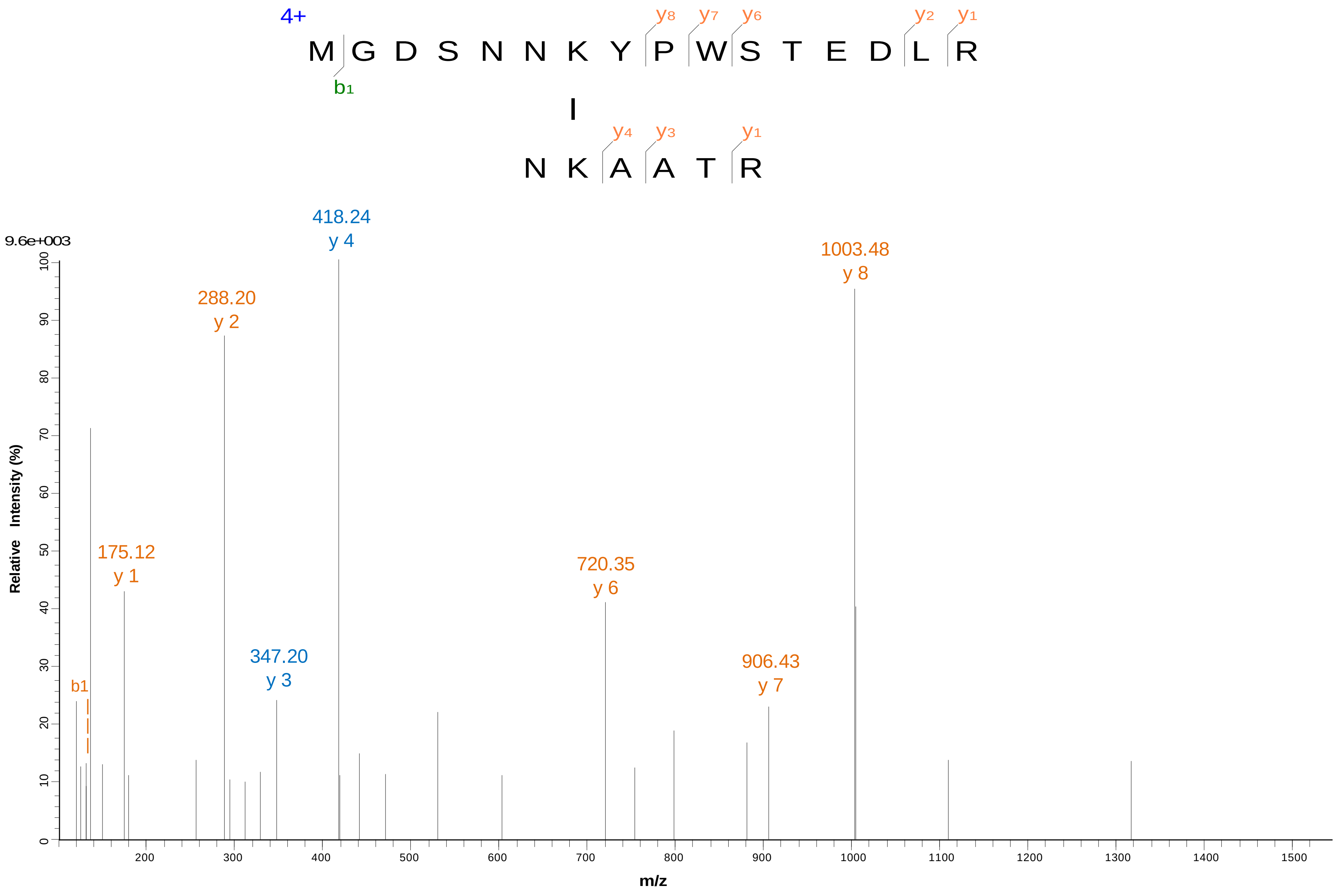
4+
M G D S N N K Y P W S T E D L R
b1
y8 y7 y6 y2 y1
N K A A T R
y4 y3 y1
9.6e+003
Relative Intensity (%)
m/z
418.24
y 4
1003.48
y 8
288.20
y 2
175.12
y 1
720.35
y 6
347.20
y 3
906.43
y 7
b1

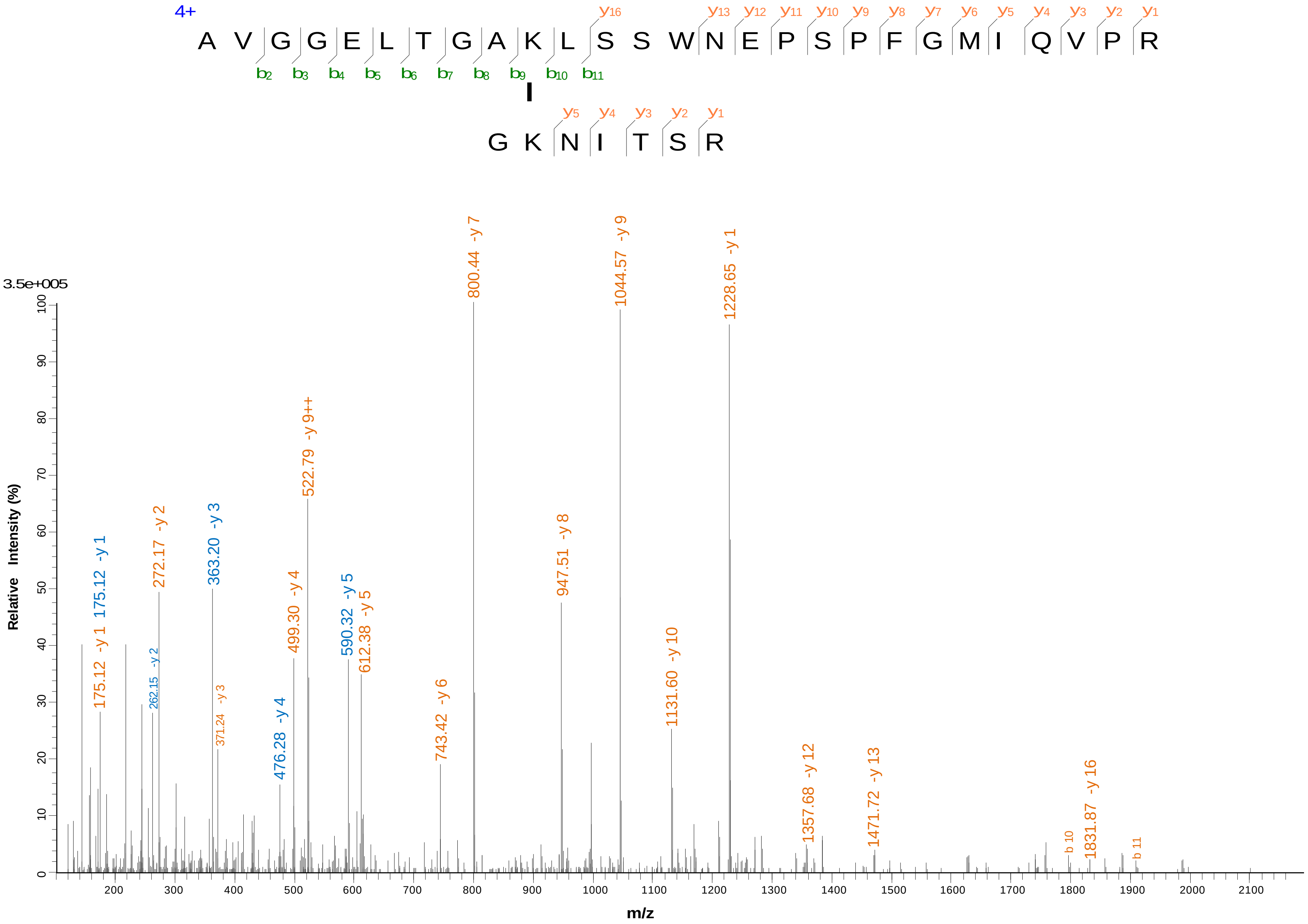

4+
A V G G E L T G A K L S S W N E P S P F G M I Q V P R
y16 y13 y12 y11 y10 y9 y8 y7 y6 y5 y4 y3 y2 y1
b2 b3 b4 b5 b6 b7 b8 b9 b10 b11
G K N I T S R
y5 y4 y3 y2 y1
3.5e+005
Relative Intensity (%)
m/z
800.44 -y 7
1044.57 -y 9
1228.65 -y 1
522.79 -y 9++
947.51 -y 8
175.12 -y 1
272.17 -y 2
363.20 -y 3
499.30 -y 4
590.32 -y 5
612.38 -y 5
175.12 -y 1
262.15 -y 2
371.24 -y 3
476.28 -y 4
743.42 -y 6
1131.60 -y 10
1357.68 -y 12
1471.72 -y 13
1831.87 -y 16
b 10
b 11

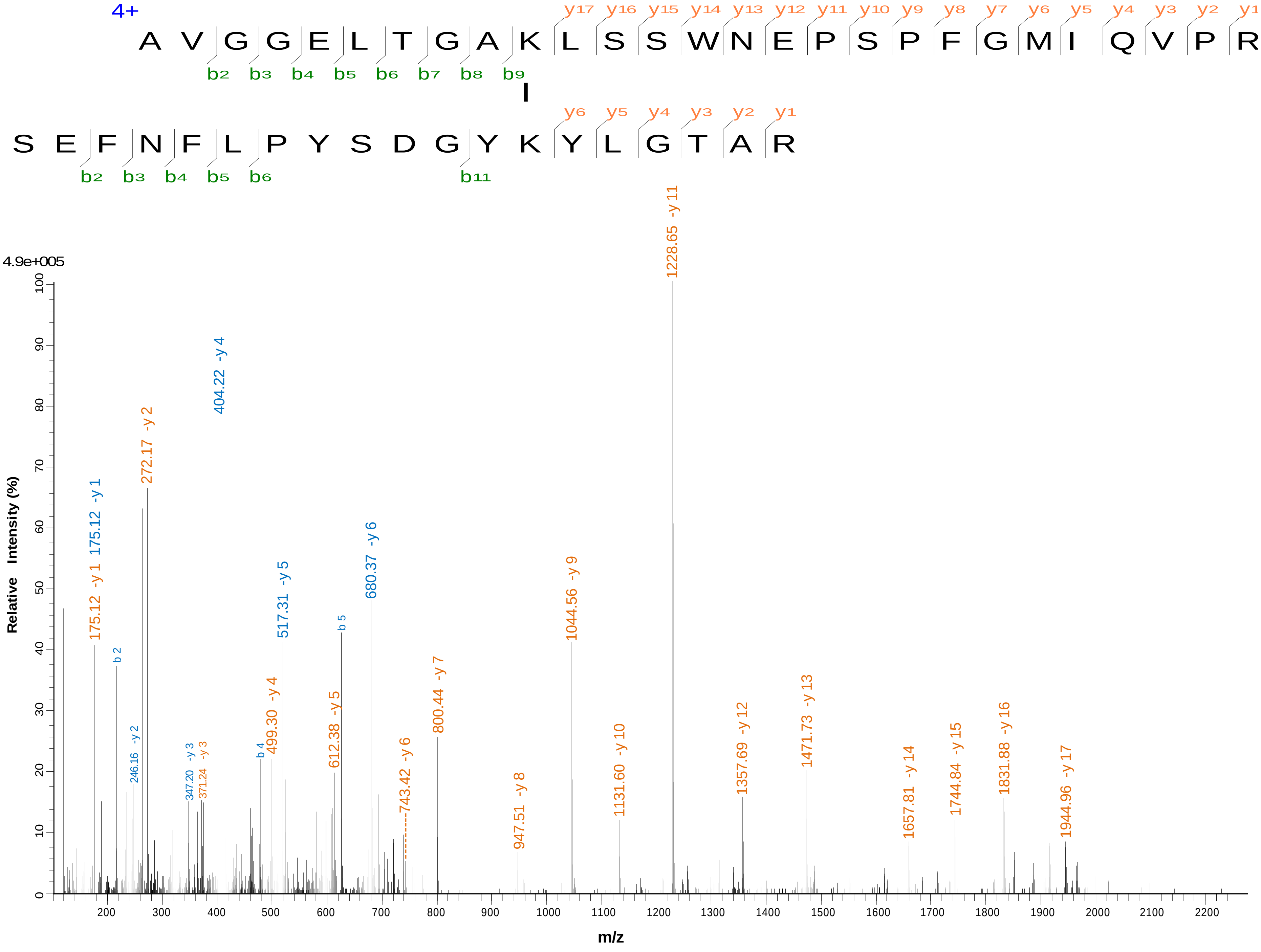
4+
A V G G E L T G A K L S S W N E P S P F G M I Q V P R
y17 y16 y15 y14 y13 y12 y11 y10 y9 y8 y7 y6 y5 y4 y3 y2 y1
b2 b3 b4 b5 b6 b7 b8 b9
S E F N F L P Y S D G Y K Y L G T A R
y6 y5 y4 y3 y2 y1
b2 b3 b4 b5 b6 b11
4.9e+005
Relative Intensity (%)
m/z
175.12 -y1
175.12 -y1
b2
246.16 -y2
272.17 -y2
347.20 -y3
371.24 -y3
404.22 -y4
b4
499.30 -y4
517.31 -y5
612.38 -y5
b5
680.37 -y6
743.42 -y6
800.44 -y7
947.51 -y8
1044.56 -y9
1131.60 -y10
1228.65 -y11
1357.69 -y12
1471.73 -y13
1657.81 -y14
1744.84 -y15
1831.88 -y16
1944.96 -y17

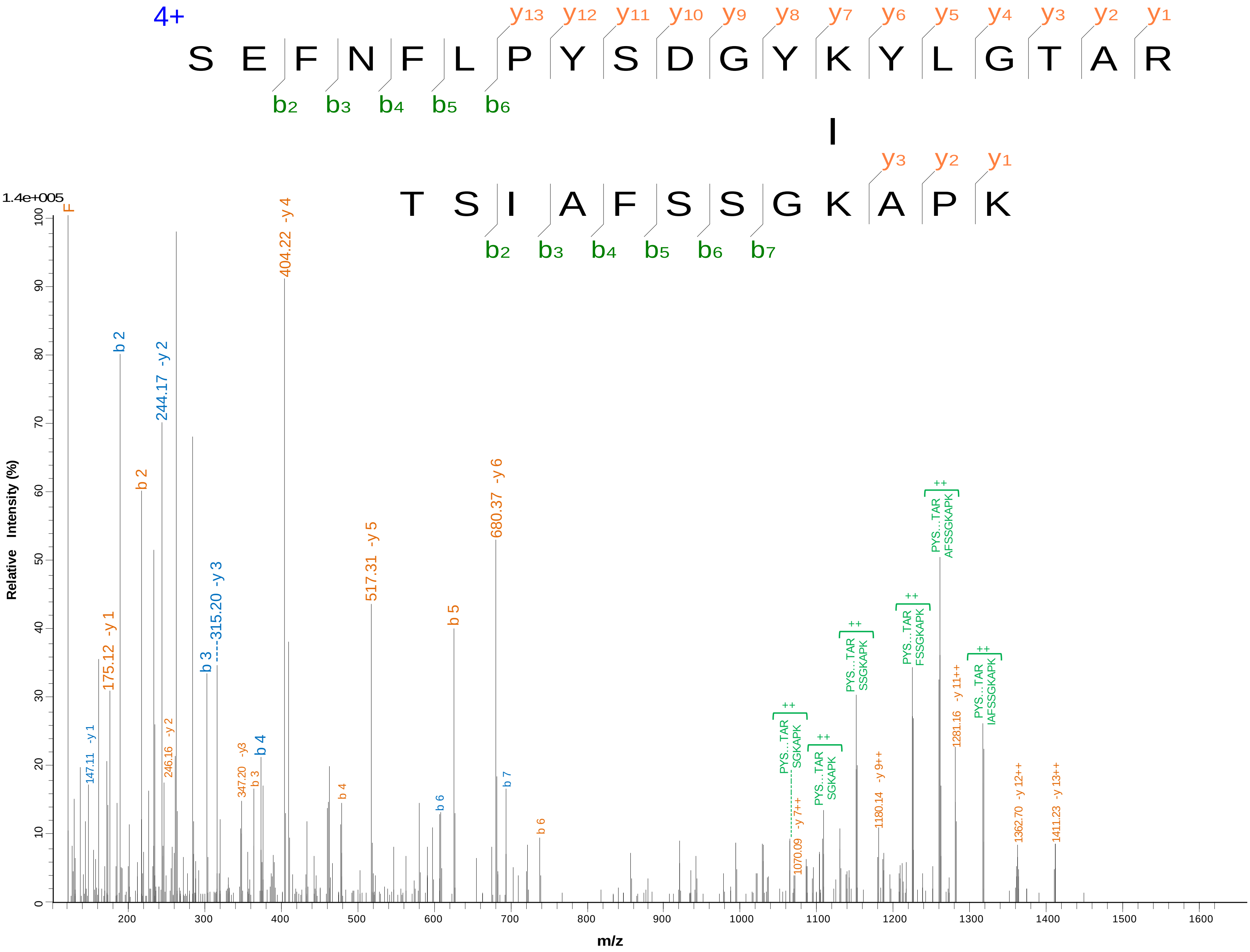

4+
S E F N F L P Y S D G Y K Y L G T A R
y13 y12 y11 y10 y9 y8 y7 y6 y5 y4 y3 y2 y1
b2 b3 b4 b5 b6
T S I A F S S G K A P K
y3 y2 y1
b2 b3 b4 b5 b6 b7
1.4e+005
Relative Intensity (%)
m/z
F
b 2
244.17 -y 2
b 2
175.12 -y 1
147.11 -y 1
246.16 -y 2
b 3
315.20 -y 3
347.20 -y3
b 3
b 4
404.22 -y 4
b 4
517.31 -y 5
b 5
b 6
680.37 -y 6
b 7
b 6
PYS...TAR SGKAPK ++
1070.09 -y 7++
PYS...TAR SGKAPK ++
PYS...TAR SSGKAPK ++
1180.14 -y 9++
PYS...TAR FSSGKAPK ++
PYS...TAR AFSSGKAPK ++
1281.16 -y 11++
PYS...TAR IAFSSGKAPK ++
1362.70 -y 12++
1411.23 -y 13++

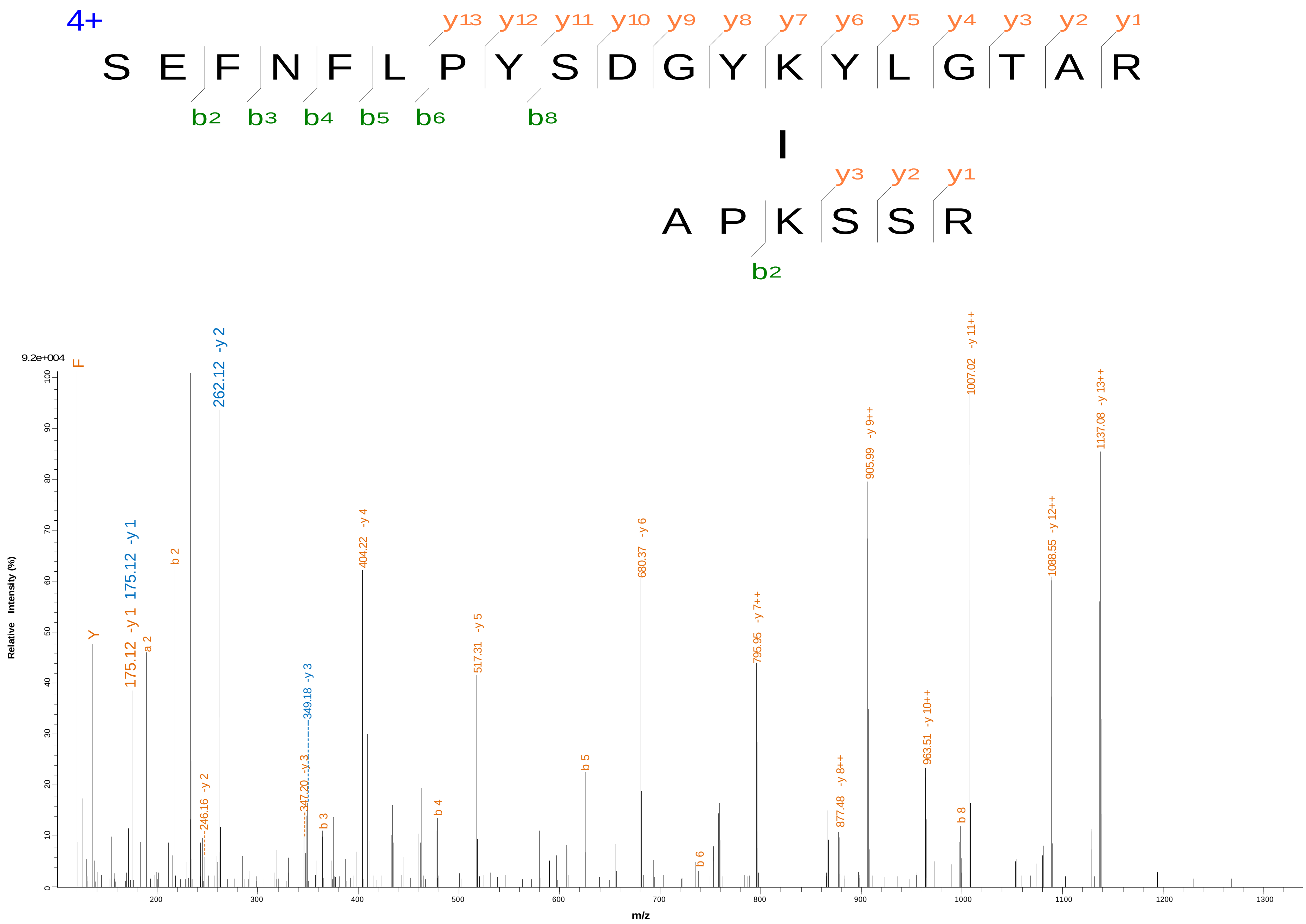

4+
y13 y12 y11 y10 y9 y8 y7 y6 y5 y4 y3 y2 y1
S E F N F L P Y S D G Y K Y L G T A R
b2 b3 b4 b5 b6 b8
y3 y2 y1
A P K S S R
b2
9.2e+004
Relative Intensity (%)
m/z
F
Y
175.12 -y 1
175.12 -y 1
a 2
b 2
262.12 -y 2
246.16 -y 2
347.20 -y 3
349.18 -y 3
b 3
404.22 -y 4
b 4
517.31 -y 5
b 5
680.37 -y 6
b 6
795.95 -y 7++
877.48 -y 8++
905.99 -y 9++
963.51 -y 10++
b 8
1007.02 -y 11++
1088.55 -y 12++
1137.08 -y 13++

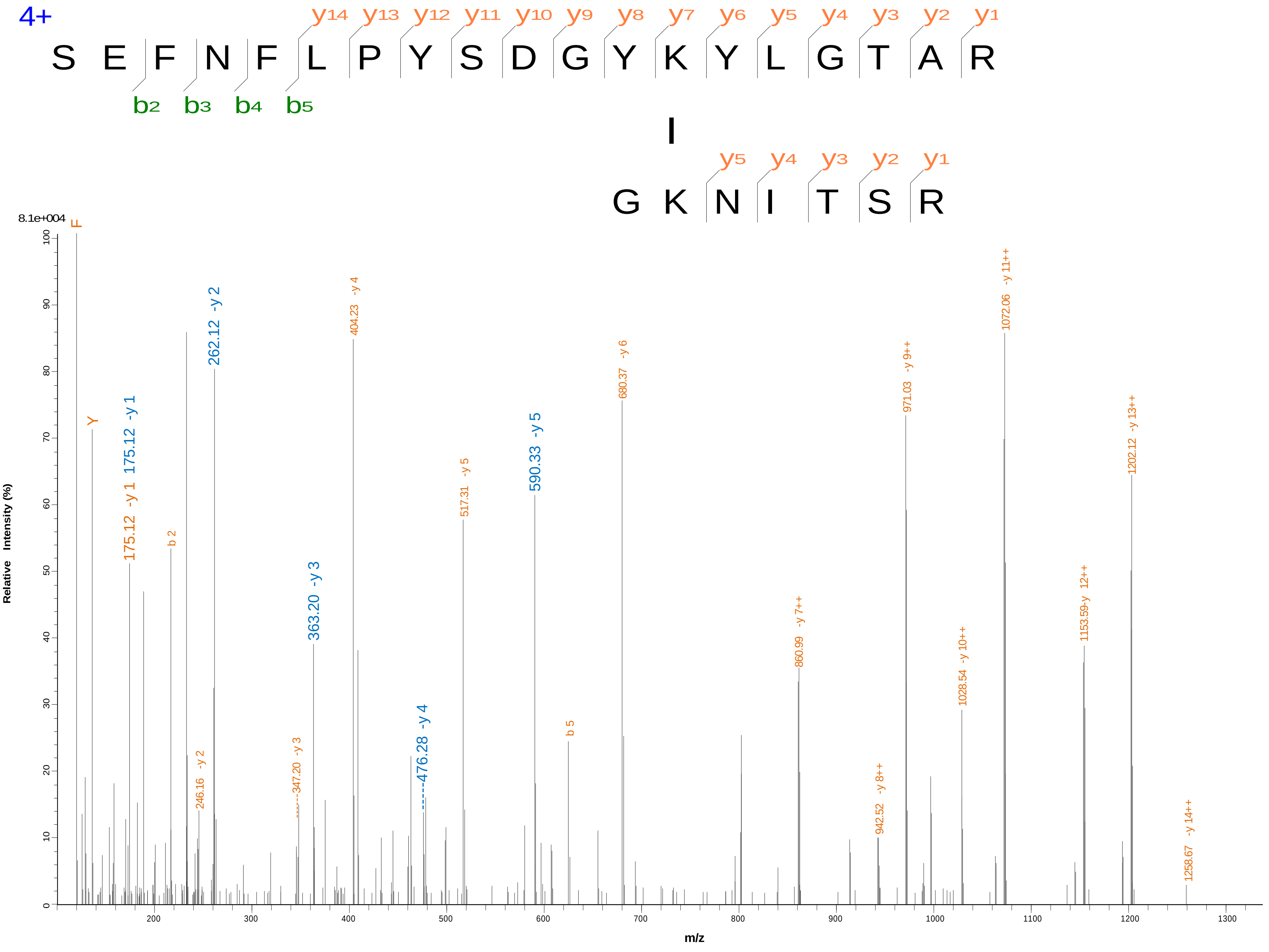
4+
y14 y13 y12 y11 y10 y9 y8 y7 y6 y5 y4 y3 y2 y1
S E F N F L P Y S D G Y K Y L G T A R
b2 b3 b4 b5
I
y5 y4 y3 y2 y1
G K N I T S R
8.1e+004
Relative Intensity (%)
m/z
F
Y
175.12 -y 1
175.12 -y 1
b 2
246.16 -y 2
262.12 -y 2
347.20 -y 3
363.20 -y 3
404.23 -y 4
476.28 -y 4
517.31 -y 5
590.33 -y 5
b 5
680.37 -y 6
860.99 -y 7++
942.52 -y 8++
971.03 -y 9++
1028.54 -y 10++
1072.06 -y 11++
1153.59-y 12++
1202.12 -y 13++
1258.67 -y 14++
