# Supplementary material for: Structural Insights into the PorK and PorN Components of the Porphyromonas gingivalis Type IX Secretion System
Source: PLoS Pathog. 2016 Aug 10;12(8):e1005820. doi: 10.1371/journal.ppat.1005820 (PMC4980022; doi:10.1371/journal.ppat.1005820)
Supplement: S3 Fig — (PDF) [file ppat.1005820.s003.pdf]

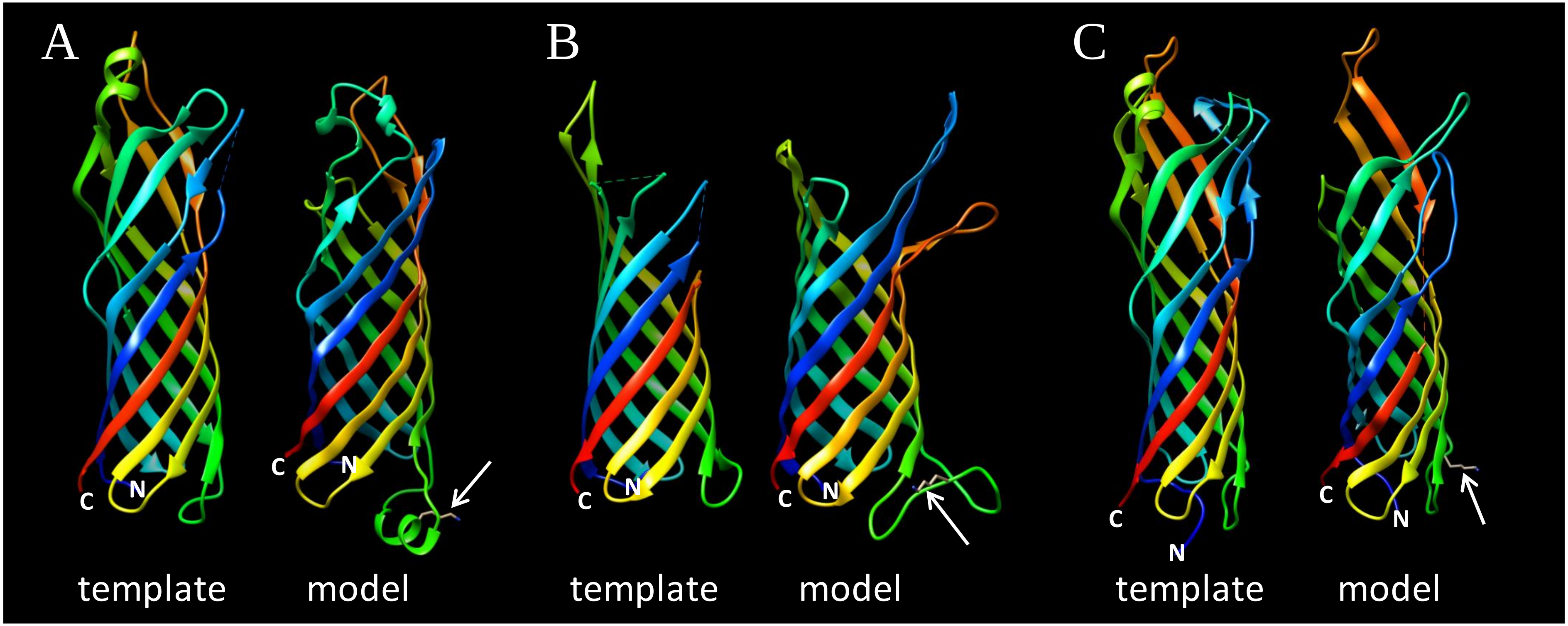

S3 Figure: Molecular modelling of PG0189 using Phyre-2. Three models are shown based on the three best matching (but different) template structures. A) OmpW full length from *E. coli*, PDB accession 2F1T; B) OmpA trans-membrane domain from *E. coli*, PDB accession 1QJP; C) OprG full length from *P. aeruginosa*, PDB accession 2X27. The N- and C-termini are shown and the cross-linked lysine residue is indicated by an arrow.
