# Supplementary material for: Structural Insights into the PorK and PorN Components of the Porphyromonas gingivalis Type IX Secretion System
Source: PLoS Pathog. 2016 Aug 10;12(8):e1005820. doi: 10.1371/journal.ppat.1005820 (PMC4980022; doi:10.1371/journal.ppat.1005820)
Supplement: S4 Fig — (PDF) [file ppat.1005820.s004.pdf]

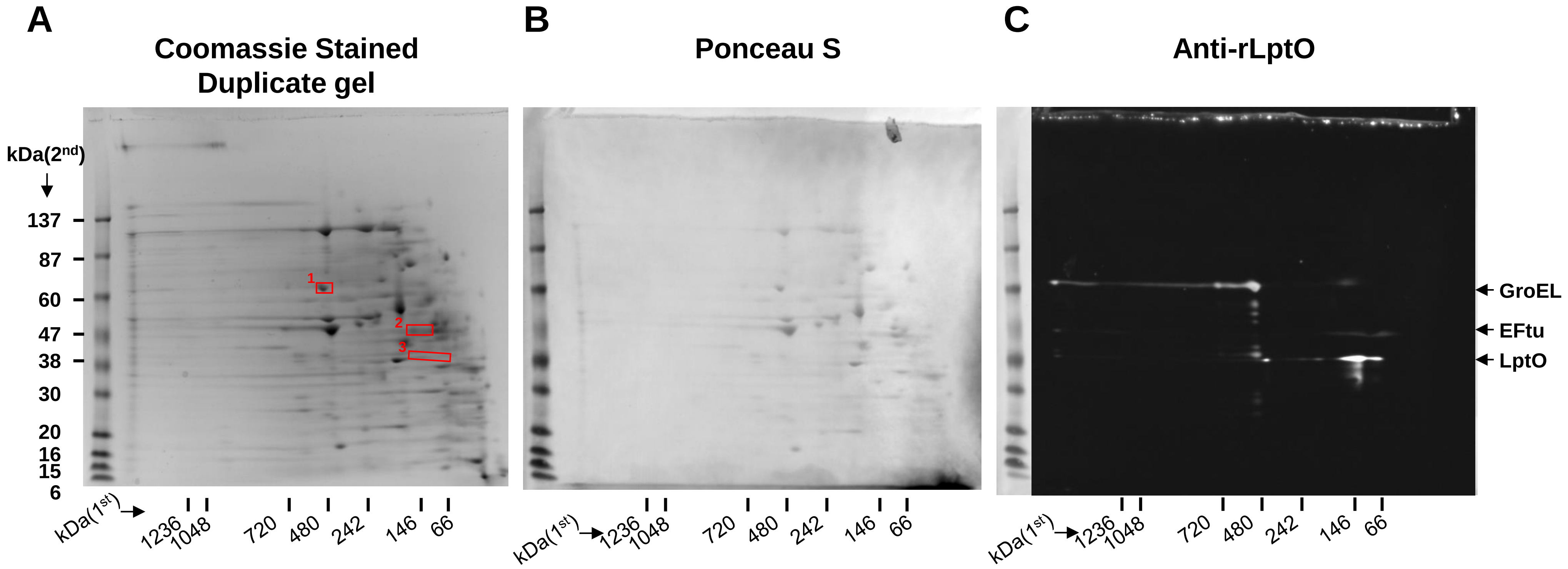

S4 Figure. Confirmation of presence of antibodies specific to *P. gingivalis* GroEL and EFtu in the anti-rLptO antisera

A. Cells from *P. gingivalis* strain 33277 were solubilized in blue native PAGE sample buffer containing 1% DDM and subjected to 2 dimensional blue native-PAGE/SDS PAGE (2D BN-PAGE) and stained with Coomassie as described in Glew et al 2014 [33]. Proteins cut for LC-MS/MS analysis (corresponding to proteins recognised by the mouse anti-rLptO serum, see panel C) are shown by red boxes and numbered. LC-MS/MS results were: 1. GroEL (equivalent to the heptameric homo-complex of GroEL) with mascot score of 1132 (top score); 2. EFtu with mascot score of 553 (top score); 3. LptO with mascot score of 340. **B**. Duplicate 2D BN-PAGE gel transferred to nitrocellulose membrane and stained with Ponceau S. **C**. Anti-rLptO Western of nitrocellulose transfer shown in **B** with second dimension location of proteins identified by antiserum indicated on right-hand-side. GroEL and EFtu are abundant proteins and are highly conserved between *E. coli* and *P. gingivalis.*
