# Supplementary material for: Structural Insights into the PorK and PorN Components of the Porphyromonas gingivalis Type IX Secretion System
Source: PLoS Pathog. 2016 Aug 10;12(8):e1005820. doi: 10.1371/journal.ppat.1005820 (PMC4980022; doi:10.1371/journal.ppat.1005820)
Supplement: S3 Table — (DOCX) [file ppat.1005820.s007.docx]

**S3 Table. High sensitivity amino acid analysis of PorK and PorN in PorK/N complexes**

**PorK**

| **Name** | **Amino acid**  **(-H_2_0) ng/sample** | **Amino acid**  **pmol/sample** | **No. of amino acids** | **Protein**  **pmol/sample ^a^** |
| --- | --- | --- | --- | --- |
| Histidine | 27 | 197 | 6 | 32.8 |
| Serine | 108 | 1245 | 41 | 30.4 |
| Arginine | 177 | 1136 | 34 | 33.4 |
| Glycine | 62 | 1081 | 31 | 34.9 |
| Aspartic acid | 202 | 1756 | 52 | 33.8 |
| Glutamic acid | 195 | 1509 | 45 | 33.5 |
| Threonine | 83 | 820 | 26 | 31.5 |
| Alanine | 78 | 1098 | 34 | 32.3 |
| Proline | 89 | 918 | 28 | 32.8 |
| Lysine | 100 | 782 | 24 | 32.6 |
| Tyrosine | 138 | 847 | 26 | 32.6 |
| Methionine | 27 | 206 | 9 | 22.9 |
| Valine | 87 | 877 | 26 | 33.7 |
| Isoleucine | 98 | 863 | 28 | 30.8 |
| Leucine | 86 | 763 | 22 | 34.7 |
| Phenylalanine | 98 | 668 | 20 | 33.4 |
| **Total** | **1656** | **14766** | **452** | **516.1** |

**PorN**

| **Name** | **Amino acid**  **(-H_2_0) ng/sample** | **Amino acid**  **pmol/sample** | **No. of amino acids** | **Protein**  **pmol/sample ^a^** |
| --- | --- | --- | --- | --- |
| Histidine | 7 | 54 | 1 | 54.0 |
| Serine | 65 | 747 | 19 | 39.3 |
| Arginine | 179 | 1147 | 29 | 39.6 |
| Glycine | 46 | 798 | 15 | 53.2 |
| Aspartic acid | 200 | 1735 | 43 | 40.3 |
| Glutamic acid | 217 | 1684 | 42 | 40.1 |
| Threonine | 92 | 914 | 25 | 36.6 |
| Alanine | 60 | 845 | 21 | 40.2 |
| Proline | 65 | 673 | 16 | 42.1 |
| Lysine | 134 | 1044 | 28 | 37.3 |
| Tyrosine | 98 | 598 | 15 | 39.9 |
| Methionine | 20 | 149 | 6 | 24.8 |
| Valine | 69 | 698 | 17 | 41.1 |
| Isoleucine | 74 | 650 | 17 | 38.2 |
| Leucine | 89 | 786 | 20 | 39.3 |
| Phenylalanine | 91 | 616 | 16 | 38.5 |
| **Total** | **1505** | **13137** | **330** | **644.5** |

**^a^** The amount of protein (pmol/sample) was calculated by dividing the amount of amino acid (pmol/sample) by the number of times that amino acid occurs in the protein sequence.

Average amount of PorK = 516.1/16 = 32.26 pmol

Average amount of PorN = 644.5/16 = 40.28 pmol

**Molar ratio PorN:PorK = 40.28/32.26 = 1.2:1**
