# Supplementary material for: Structural Insights into the PorK and PorN Components of the Porphyromonas gingivalis Type IX Secretion System
Source: PLoS Pathog. 2016 Aug 10;12(8):e1005820. doi: 10.1371/journal.ppat.1005820 (PMC4980022; doi:10.1371/journal.ppat.1005820)
Supplement: S7 Table — (DOCX) [file ppat.1005820.s011.docx]

S7 Table: Species distribution of PG0189 homologs

| Species and strain | Protein Description | GenBank Accession | Score (bits) | Query Cover (%) | Expect value | Identity (%) | Taxonomy Class |
| --- | --- | --- | --- | --- | --- | --- | --- |
| *Parabacteroides merdae* ATCC 43184 | hypothetical protein PARMER_01447 | EDN87112 | 299 | 98 | 2E-100 | 41 | Bacteroidia |
| *Prevotella intermedia* 17 | outer membrane protein beta-barrel domain protein PIN17_A0684 | AFJ08348 | 289 | 96 | 2E-96 | 30 | Bacteroidia |
| *Tannerella forsythia* ATCC 43037 | hypothetical protein Tanf_09815 | KKY60995 | 260 | 96 | 6E-85 | 40 | Bacteroidia |
| *Bacteroides thetaiotaomicron* VPI-5482 | major outer membrane protein OmpA | AAO77592 | 54.4 | 93 | 2E-05 | 18 | Bacteroidia |
| *Bacteroides fragilis* NCTC 9343 | putative outer membrane protein | CAH07002 | 52.1 | 70 | 1E-04 | 21 | Bacteroidia |
| *Cytophaga hutchinsonii* ATCC 33406 | conserved hypothetical protein CHU_3410 | ABG60646 | 158 | 95 | 4E-45 | 21 | Cytophagia |
| *Flavobacterium johnsoniae* UW101 | hypothetical protein Fjoh_1692 | ABQ04724 | 165 | 91 | 6E-48 | 18 | Flavobacteriia |
| *Chitinophaga pinensis* DSM 2588 | hypothetical protein Cpin_3365 | ACU60832 | 134 | 96 | 5E-35 | 16 | Chitinophagia |
| *Pedobacter heparinus* DSM 2366 | hypothetical protein Phep_0984 | ACU03206 | 153 | 91 | 8E-43 | 22 | Sphingobacteriia |

This table summarises the key findings from a PSI-BLAST search (2 iterations) of PG0189. Homologs were found within several distantly related species belonging to different taxonomic classes. These species are known to have the T9SS. In contrast, only distantly related homologs were found in the closely related species, *B. thetaiotaomicron* and *B. fragilis* which do not have the T9SS. These two homologs are significantly larger than PG0189 and are actually more closely related to Omp40 (PG0694) and Omp41 (PG0695), with expect values less than 1E-27 after a single psi-BLAST iteration. The results are therefore consistent with PG0189 being a component of the T9SS. Omp40 and Omp41 are also OmpA-like proteins and therefore their relatedness to PG0189 is consistent with the Phyre-2 results.
